# Inference for microbe–metabolite association networks using a latent graph model

**DOI:** 10.1093/biomtc/ujag042

**Published:** 2026-03-10

**Authors:** Jing Ma

**Affiliations:** Division of Public Health Sciences, Fred Hutchinson Cancer Center, Seattle, WA 98109, United States

**Keywords:** bipartite stochastic block model, false discovery rate, microbiome multi-omics, network analysis, power

## Abstract

Correlation networks are commonly used to infer associations between microbes and metabolites. The resulting $p$-values are then corrected for multiple comparisons using existing methods such as the Benjamini & Hochberg (BH) procedure to control the false discovery rate (FDR). However, most existing methods for FDR control assume the $p$-values are weakly dependent. Consequently, they can have low power in recovering microbe–metabolite association networks that exhibit important topological features, such as the presence of densely associated modules. We propose a novel inference procedure that is both powerful for detecting significant associations in the microbe–metabolite network and capable of controlling the FDR. Power enhancement is achieved by modeling latent structures in the form of a bipartite stochastic block model. We develop a variational expectation–maximization (EM) algorithm to estimate the model parameters and incorporate the learned graph in the testing procedure. In addition to FDR control, this procedure provides a clustering of microbes and metabolites into modules, which is useful for interpretation. We demonstrate the merit of the proposed method in simulations and an application to bacterial vaginosis.

## 1 Introduction

The gut microbiome plays an important role in human health and diseases (Hou et al., 2022). One potential pathway by which the gut microbiome impacts host health is through microbial-derived metabolites: these molecules can modulate the immune system (Dinglasan et al., 2025; Williams and Cao, 2024). Yet, despite extensive experimental and computational work, a large number of microbe–metabolite relationships remain unknown, leaving a significant knowledge gap (Liu et al., 2022).

Our motivating example concerns a study of bacterial vaginosis (BV) using paired microbiome and metabolome profiling (McMillan et al., 2015). The vaginal microbiota is an intricate ecosystem in which microbes trade nutrients, defense molecules, and signaling compounds with both the host epithelium and one another. Disruption of this ecosystem manifests clinically as BV, a condition affecting up to 30% of women worldwide (Koumans et al., 2007) and linked to adverse outcomes such as pre-term birth and increased risk to sexually transmitted infections (Guerra et al., 2006; Atashili et al., 2008). A growing body of evidence suggests that BV is not driven by a single pathogen but reflect a community-wide metabolic shift (Srinivasan et al., 2015; Łaniewski and Herbst-Kralovetz, 2021). Improved diagnosis and treatment of BV requires a detailed understanding of these metabolic activities.

The data set contains 49 taxa and 128 metabolites collected from 124 individuals. For the $i$-th taxon and the $j$-th metabolite, a correlation statistic and its $p$-value can be derived. The task is to perform simultaneous inference for the null hypothesis of no association between all taxa and metabolites, for $i=1,\ldots ,49$ and $j=1,\ldots ,128$.

The goal of large-scale hypothesis testing is to identify as many interesting ones as possible while keeping the error rate low. The false discovery rate (FDR), defined as the average proportion of errors among the discoveries, is a commonly used metric to assess the significance of testing multiple hypotheses simultaneously. In practice, the Benjamini & Hochberg (BH) procedure based on ranking $p$-values (Benjamini and Hochberg, 1995) is often used to control FDR. While easy to implement, this vector-based approach suffers from two main limitations. First, it provides a list of significant associations that can be hard to interpret biologically. Second, BH and related methods such as Storey’s $q$-value (Storey et al., 2004) assume weakly dependent $p$-values; this assumption is often violated when analyzing high-dimensional microbiome and metabolomic data, especially when microbes and metabolites form densely connected modules.

For vector-based testing problems, many authors have improved power by estimating the null/alternative mixture distributions (Efron, 2004; Sun and Cai, 2007) and modeling the dependence explicitly (Sun and Cai, 2009; Li et al., 2024; Wang and Wang, 2024). Those advances, however, do not translate directly to matrix-valued data such as correlation or cross-correlation matrices: here the tests exhibit structured dependence that is not exploited by existing tools. In large-scale testing of (partial) correlations, Liu (2013), Cai and Liu (2016), Xia et al. (2015) and Cai et al. (2019) established valid FDR control for test statistics obtained from high-dimensional data under mild regularity conditions, but they do not take into account the structural information in the data.

Rebafka et al. (2022) recently bridged this gap with the *noisy stochastic block model* (noisySBM), which treats the constellation of null and non-null hypotheses itself as an SBM and models the observed statistics as a perturbed version of that latent graph. Applied naively to bipartite data, however, the standard noisySBM (i) is computationally heavy and (ii) tends to merge features of different types into the same community (Larremore et al., 2014), degrading FDR control.

We introduce a bipartite noisySBM that models the hidden microbe–metabolite interaction network as a bipartite SBM and embeds it in the observed statistics through a two-component mixture of the null and alternative densities. The proposed framework offers three key advances. First, separate block memberships are learned for microbes and metabolites, respecting their asymmetry and avoiding the mixing issue of the standard SBM. These inferred modules are useful for biological interpretation. Second, within each block the method pools information to compute structured $\ell$-values which are the posterior of a null hypothesis being true given observed data and the latent graph. Thresholding these quantities controls the (marginal) FDR at the nominal level while markedly boosting the true discovery rate. Lastly, we develop a variational expectation-maximization algorithm to estimate the model parameters. This algorithm solves two smaller, type-specific sub-problems, delivering order-of-magnitude speed-ups over the original noisySBM without sacrificing accuracy. Through extensive simulations we show that the bipartite noisySBM achieves superior FDR control and power compared to existing methods. We then apply the method to paired microbiome and metabolomic data from McMillan et al. (2015). The analysis uncovers three distinct groups of taxa–metabolite associations between healthy and BV individuals. Metabolites previously linked to BV, including *2-hydroxyisovalerate (2HV), succinate*, and *GHB*, also displayed altered associations with multiple taxa. These findings illustrate how the proposed method can detect shifts in microbial–metabolite network structure and highlight taxa–metabolite relationships that may warrant further study.

The rest of the paper is organized as follow. Section 2 introduces the bipartite noisySBM and the multiple testing procedure. Section 3 describes the algorithm and model selection strategy. Section 4 benchmarks the proposed method on simulated data and Section 5 applies it to BV multi-omics data. We close with a discussion in Section 6.

## 2 Bipartite graph inference

### 2.1 Noisy bipartite stochastic block model

Let $n_1\ge 2$ be the number of microbes and $n_2\ge 2$ the number of metabolites. We observe ${\bf X}=(x_{ij})\in {\mathbb {R}}^{n_1\times n_2}$, for which each $x_{ij}$ corresponds to an association score between the $i$-th microbe and the $j$-th metabolite. We record the trueness/falseness of the null hypothesis of no association between each pair of features in a bipartite adjacency matrix ${\bf A}\in \lbrace 0,1\rbrace ^{n_1\times n_2}$, such that $A_{ij} = 0$ if and only if the corresponding null hypothesis is true. In other words, we consider simultaneously testing


\begin{eqnarray*}
H_{0,ij}: A_{ij} = 0\quad \mbox{versus.} \quad H_{1,ij}: A_{ij} = 1,
\end{eqnarray*}

for all $1\le i \le n_1$ and $1\le j \le n_2$.

We model ${\bf A}$ with a bipartite stochastic block model characterized by two independent membership vectors ${\bf Z}_1=(Z_{i,1})_{1\le i\le n_1}$ and ${\bf Z}_2=(Z_{j,2})_{1\le j\le n_2}$ where


\begin{eqnarray*}
Z_{i,1}\sim \mathrm{Multinomial}\bigl (1,\boldsymbol{\alpha }_1\bigr ),\qquad Z_{j,2}\sim \mathrm{Multinomial}\bigl (1,\boldsymbol{\alpha }_2\bigr ).
\end{eqnarray*}

The mixing proportions $\boldsymbol{\alpha }_1=(\alpha _{q,1})_{1\le q\le B_1}$ and $\boldsymbol{\alpha }_2=(\alpha _{l,2})_{1\le q\le B_2}$ satisfy $\sum _{q=1}^{{B_1}}\alpha _{q,1} = 1$ and $\sum _{q=1}^{{{B_2}}}\alpha _{q,2} = 1$. Conditional on the membership vectors, the edges are independent Bernoulli variables with


\begin{eqnarray*}
A_{ij}\,\,|\,\,{\bf Z}_1,{\bf Z}_2\,\,\sim \,\,\mathrm{Bernoulli}\bigl (\pi _{Z_{i,1},\, Z_{j,2}}\bigr ), \, \boldsymbol{\Pi }=(\pi _{ql})\in (0,1)^{B_1\times B_2}.
\end{eqnarray*}

For each pair $(i,j)$ the test statistic $x_{ij}$ is modeled as a two-component mixture:


\begin{eqnarray*}
x_{ij}\, \bigl |\, {\bf A},{\bf Z}_1,{\bf Z}_2 \sim \left\lbrace \begin{array}{@{}l@{\quad }l@{}}g_0(\, \cdot \, ;\nu _0), & A_{ij}=0,\\
g(\, \cdot \, ;\nu _{Z_{i,1},\, Z_{j,2}}), & A_{ij}=1, \end{array}\right.
\end{eqnarray*}

where $g_0(\cdot ; \nu _0)$ and $g(\cdot ; \nu )$ are pre-specified null and alternative densities with parameters $\nu _0\subset {\cal V}_0$ and $\nu \subset {\cal V}$. Both families are parametric with ${\cal V}_0 \subset \mathbb {R}^{d_0}$ and ${\cal V}\subset \mathbb {R}^{d_1}$, where $d_0$ and $d_1$ are the respective dimensions of the parameter spaces. The density functions $g_0$ and $g$ are usually Gaussian but can take other forms. The complete parameter set is $\boldsymbol{\theta }=(\boldsymbol{\alpha }_1,\boldsymbol{\alpha }_2,\boldsymbol{\Pi },\nu _0,\nu )$.

The formulation reduces to the noisySBM when $B_1\!=\!B_2$ and rows/columns share a single clustering, but the bipartite version avoids the tendency of a standard SBM to *merge* different vertex types into the same community and greatly improves both speed and accuracy for bipartite data (Larremore et al., 2014). In general, the number of row clusters ${B_1}$ and the number of column clusters ${{B_2}}$ do not have to be the same (Yen and Larremore, 2020).

### 2.2 Identifiability

Before we discuss the testing procedure and model estimation, we address the model’s identifiability issue. To this end, we introduce the following assumptions.

Assumption 1: The parameters $\lbrace \nu _0\subset {\cal V}_0 \subset {\cal V}, \nu _{ql}\subset {\cal V}: 1\le q \le B_1, 1\le l \le B_2\rbrace$ are distinct.

Assumption 2: The parameters of any finite mixture of distributions $\lbrace g(\cdot ; \nu ): \nu \subset {\cal V}\rbrace$ are identifiable, up to label swapping.

Assumption 2 is satisfied for the class of Gaussian densities that often arises in hypothesis testing.

Theorem 1: Let $n_1,n_2\ge 2$ and $B_1,B_2\ge 2$. Let $g_0(\cdot ;\nu )$ and $g(\cdot ;\nu )$ be from the same family of distributions. Under Assumptions 1-2, all parameters of the bipartite noisy stochastic block model are identifiable, up to label swapping.

The proof of Theorem 1 can be adapted from the proofs in Allman et al. (2011) and Rebafka et al. (2022), and is thus omitted. The key modification required is to replace the triangle $(X_{ij},X_{jk},X_{ki})$ with the smallest fully connected subgraph $(X_{i_1 j_1},X_{i_1 j_2},X_{i_2 j_1}, X_{i_2 j_2})$. Just as the triangle is “minimal” for the unipartite SBM, the 2 by 2 square is minimal for the bipartite case: four edges are enough to identify $\nu _0, \nu _{ql}$ and the mixing proportions $\boldsymbol{\alpha }_1, \boldsymbol{\alpha }_2$. When $g(\cdot ,\nu _0)$ and $g(\cdot ,\nu )$ are Gaussian densities, the model is identifiable provided the parameter pairs $(\mu _{ql},\sigma _{ql}^2)$ and $(0,\sigma _0^2)$ are all distinct. More generally, identifiability still holds if a single alternative density is shared across blocks ($\mu _{ql}=\mu ,\,\,\sigma ^2_{ql}=\sigma ^2$).

### 2.3 Graph-based multiple-testing

Let $\varphi ({\bf X})\in \lbrace 0,1\rbrace ^{{\bf A}}$ be a decision rule that rejects $H_{0,ij}$ when $\varphi _{ij}({\bf X})=1$. Under parameter $\boldsymbol{\theta }$ we evaluate the false discovery rate (FDR), true discovery rate (TDR), and marginal FDR (mFDR) defined by


\begin{eqnarray*}
\mathrm{FDR}_{\boldsymbol{\theta }}(\varphi ) &=& \mathbb {E}_{\boldsymbol{\theta }}\!\left[ \frac{\sum _{(i,j)}(1-A_{ij})\varphi _{ij}({\bf X})}{\bigl \lbrace \sum _{(i,j)}\varphi _{ij}({\bf X})\bigr \rbrace \vee 1} \right], \\
\mathrm{TDR}_{\boldsymbol{\theta }}(\varphi ) &=& \frac{\mathbb {E}_{\boldsymbol{\theta }}\bigl [\sum _{(i,j)}A_{ij}\varphi _{ij}({\bf X})\bigr ]}{\mathbb {E}_{\boldsymbol{\theta }}\bigl [\sum _{(i,j)}A_{ij}\bigr ]},
\end{eqnarray*}

\begin{eqnarray*}
\mathrm{mFDR}_{\boldsymbol{\theta }}(\varphi ) = \frac{\mathbb {E}_{\boldsymbol{\theta }}\bigl [\sum _{(i,j)}(1-A_{ij})\varphi _{ij}({\bf X})\bigr ]}{\mathbb {E}_{\boldsymbol{\theta }}\bigl [\sum _{(i,j)}\varphi _{ij}({\bf X})\bigr ]}.
\end{eqnarray*}

The mFDR is easier to estimate because it involves the ratio of expectations as opposed to the expectation of a ratio; for large numbers of tests it is asymptotically equivalent to the FDR (Genovese and Wasserman, 2002).

Given a target level $\alpha$, we seek a rule that maximizes TDR subject to $\mathrm{mFDR}_{\boldsymbol{\theta }}\le \alpha$. Define the *structured $\ell$-value*


\begin{eqnarray*}
\ell (x_{ij};{\bf Z}_1,{\bf Z}_2,\boldsymbol{\theta })=P\bigl (A_{ij}=0\mid {\bf X},{\bf Z}_1,{\bf Z}_2;\boldsymbol{\theta }\bigr ) = \ell \bigl (x_{ij},Z_{i,1},Z_{j,2};\boldsymbol{\theta }\bigr ),
\end{eqnarray*}

where, for block $(q,\ell )$,


\begin{eqnarray*}
\ell (x_{ij},q,\ell ;\boldsymbol{\theta })= \frac{(1-\pi _{ql})\, g_0(x_{ij};\nu _0)}{\pi _{ql}\, g(x_{ij};\nu _{ql}) + (1-\pi _{ql})\, g_0(x_{ij};\nu _0)}.
\end{eqnarray*}

Unlike a classical BH $p$-value, the $\ell$-value pools information across *all* observations via the shared parameters and cluster memberships, leading to substantial power gains.

In practice $({\bf Z}_1,{\bf Z}_2,\boldsymbol{\theta })$ are unknown. Let $(\widehat{{\bf Z}}_1,\widehat{{\bf Z}}_2,\widehat{\boldsymbol{\theta }})$ denote their estimates obtained using the algorithm discussed in Section 3. The hypothesis $H_{0,ij}$ is rejected whenever


\begin{eqnarray*}
\ell \bigl (x_{ij};\widehat{{\bf Z}}_1,\widehat{{\bf Z}}_2,\widehat{\boldsymbol{\theta }}\bigr )\le \tau ,
\end{eqnarray*}

where $\tau =\tau (\alpha )$ is the largest threshold for which the plug-in estimate of mFDR does not exceed $\alpha$.

## 3 Parameter estimation

Estimating the model parameters $\boldsymbol{\theta }=\!(\boldsymbol{\alpha }_1,\boldsymbol{\alpha }_2,\boldsymbol{\Pi },\nu _0,\nu )$ from the observed data matrix ${\bf X}=(x_{ij})$ is challenging, because the likelihood must be integrated over the unobserved adjacency matrix ${\bf A}$ and the block-membership vectors $({\bf Z}_1,{\bf Z}_2)$. In addition, the conditional distribution of the latent variables (${\bf A},{\bf Z}_1,{\bf Z}_2$) given observed data does not have a closed-form expression. We therefore maximize a variational lower bound on the marginal likelihood via a *variational expectation–maximization* (VEM) algorithm.

### 3.1 Variational EM algorithm

For any distribution $Q$ on $({\bf A},{\bf Z}_1,{\bf Z}_2)$, the observed data log-likelihood can be decomposed as


(1) \begin{eqnarray*}
\log L({\bf X};\boldsymbol{\theta })&=& \mbox{E}_Q\bigl [\log L({\bf X},{\bf A},{\bf Z}_1,{\bf Z}_2;\boldsymbol{\theta })\bigr ] \,\,+\,\,H(Q) \\
&&+\mathrm{KL}\bigl (Q\, \Vert \, P_{{\bf A},{\bf Z}_1,{\bf Z}_2\mid {\bf X};\boldsymbol{\theta }}\bigr ),
\end{eqnarray*}

where $H(Q)$ is the entropy and $\mathrm{KL}(\cdot \Vert \cdot )$ denotes Kullback–Leibler divergence. Because the KL term is non-negative, the first two terms form an *evidence lower bound* (ELBO) on $\log L({\bf X};\boldsymbol{\theta })$.

The exact posterior $P_{{\bf A},{\bf Z}_1,{\bf Z}_2\mid {\bf X};\boldsymbol{\theta }}$ is intractable due to the complex structure of our model, so we restrict $Q$ to the factorized family


\begin{eqnarray*}
Q_{\boldsymbol{\beta }_1,\boldsymbol{\beta }_2}({\bf A},{\bf Z}_1,{\bf Z}_2)\,\,=\,\, P({\bf A}\mid {\bf Z}_1,{\bf Z}_2,{\bf X};\boldsymbol{\theta }) \prod _{i=1}^{n_1}\!\beta _{i,Z_{i,1},1} \prod _{j=1}^{n_2}\!\beta _{j,Z_{j,2},2},
\end{eqnarray*}

with variational parameters $\boldsymbol{\beta }_r=(\beta _{i,q,r})\in [0,1]^{n_r\times B_r}$ satisfying $\sum _{q=1}^{B_r}\beta _{i,q,r}=1$ for $r=1,2$.

#### E-step (update of $\boldsymbol{\beta }_1,\boldsymbol{\beta }_2$)

Keeping $\boldsymbol{\theta }$ fixed at its current estimate $\boldsymbol{\theta }^{(t)}$, we choose $(\boldsymbol{\beta }_1^{(t+1)},\boldsymbol{\beta }_2^{(t+1)})$ to maximize the ELBO. This is equivalent to minimizing the KL divergence in (1) and yields the fixed-point updates (see Web Appendix A)


\begin{eqnarray*}
&&\beta _{i,q,1}\,\,\propto \,\, \alpha _{q,1}\, \exp \Bigl \lbrace \! \sum _{j=1}^{n_2}\sum _{\ell =1}^{B_2} \beta _{j,\ell ,2}\, d_{ij}^{ql} \Bigr \rbrace , \\
&& \beta _{j,\ell ,2}\,\,\propto \,\, \alpha _{\ell ,2}\, \exp \Bigl \lbrace \! \sum _{i=1}^{n_1}\sum _{q=1}^{B_1} \beta _{i,q,1}\, d_{ij}^{ql} \Bigr \rbrace ,
\end{eqnarray*}

where


\begin{eqnarray*}
d_{ij}^{ql}\,\,=\,\, \rho _{ij}^{ql}\, \log \!\frac{\pi _{ql}\, g_{\nu _{ql}}(x_{ij})}{\rho _{ij}^{ql}} +(1-\rho _{ij}^{ql})\, \log \!\frac{(1-\pi _{ql})\, g_{\nu _0}(x_{ij})}{1-\rho _{ij}^{ql}}
\end{eqnarray*}

and $\rho _{ij}^{ql}=P(A_{ij}=1\mid Z_{i,1}=q,Z_{j,2}=\ell ,{\bf X};\boldsymbol{\theta }^{(t)})$. In practice, 3–5 inner iterations suffice for convergence of the fixed-point updates.

#### M-step (update of $\boldsymbol{\theta }$)

Assume for the moment the null and alternative densities are Gaussian. With $(\boldsymbol{\beta }_1,\boldsymbol{\beta }_2)$ fixed, maximizing the ELBO gives closed-form updates of $\boldsymbol{\theta }$:


\begin{eqnarray*}
\hat{\alpha }_{q,1}&=& \frac{1}{n_1}\sum _{i=1}^{n_1}\beta _{i,q,1},\qquad \hat{\alpha }_{\ell ,2}= \frac{1}{n_2}\sum _{j=1}^{n_2}\beta _{j,\ell ,2}, \\
&& \hat{\pi }_{ql}= \frac{\sum _{i,j}\beta _{i,q,1}\beta _{j,\ell ,2}\, \rho _{ij}^{ql}}{\sum _{i,j}\beta _{i,q,1}\beta _{j,\ell ,2}},
\end{eqnarray*}

\begin{eqnarray*}
\hat{\nu }_0:\,\, \sigma _0^{2}= \frac{\sum _{i,j}\!x_{ij}^{2} \sum _{q,\ell }\beta _{i,q,1}\beta _{j,\ell ,2}(1-\rho _{ij}^{ql})}{\sum _{i,j}\sum _{q,\ell } \beta _{i,q,1}\beta _{j,\ell ,2}(1-\rho _{ij}^{ql})},
\end{eqnarray*}

\begin{eqnarray*}
\hat{\nu }_{ql}:\,\, \mu _{ql}&=& \frac{\sum _{i,j}\beta _{i,q,1}\beta _{j,\ell ,2}\rho _{ij}^{ql}x_{ij}}{\sum _{i,j}\beta _{i,q,1}\beta _{j,\ell ,2}\rho _{ij}^{ql}},\quad \sigma _{ql}^{2} \\
&=& \frac{\sum _{i,j}\beta _{i,q,1}\beta _{j,\ell ,2}\rho _{ij}^{ql}(x_{ij}-\mu _{ql})^{2}}{\sum _{i,j}\beta _{i,q,1}\beta _{j,\ell ,2}\rho _{ij}^{ql}}.
\end{eqnarray*}

Updates for other tractable null and alternative densities, e.g., Gamma, can be derived similarly.

The E- and M-steps are alternated until the algorithm converges. Finally, the estimated memberships $\widehat{{\bf Z}}_r=\arg \max _q\beta _{i,q,r} \ (r=1,2)$ provide an interpretable biclustering of microbes and metabolites.

#### 3.1.1 Initialization

We initialize parameters in the algorithm in the following order: $\boldsymbol{\beta }_r, \boldsymbol{\rho }, \boldsymbol{\Pi }, \nu _0,$  $\nu$ and $\boldsymbol{\rho }$ again before initiating the M-step of the algorithm. To initialize $\boldsymbol{\beta }_r$, we use k-means clustering on the rows and columns. We threshold the observed statistics at $p$-value 0.5 to obtain a rough estimate of $\boldsymbol{\rho }=(\rho _{ij}^{ql})$ so that we can initialize $\boldsymbol{\Pi }$. The parameters $\nu _0$ and $\nu$ are derived conditional on the initial cluster memberships. Finally, we improve the initialization of $\boldsymbol{\rho }$ conditional on the initial cluster memberships, $\boldsymbol{\Pi }, \nu _0$ and $\nu$.

### 3.2 Selecting the number of clusters

The VEM algorithm requires knowing the row and column block counts $(B_1,B_2)$. We choose them by maximizing the *integrated classification likelihood (ICL)*:


\begin{eqnarray*}
\operatorname{ICL}(B_1,B_2)= \mbox{E}_{\widehat{Q}}\bigl [\log L({\bf X},{\bf A},{\bf Z}_1,{\bf Z}_2;\hat{\boldsymbol{\theta }})\bigr ] -\operatorname{pen}_{\mathrm{BIC}}(B_1,B_2),
\end{eqnarray*}

where $\widehat{Q}$ is the fitted variational distribution for the given pair $(B_1,B_2)$, $\hat{\boldsymbol{\theta }}$ the associated parameter estimate, and


\begin{eqnarray*}
&&\operatorname{pen}_{\mathrm{BIC}}(B_1,B_2)= (B_1-1)\log n_1+(B_2-1)\log n_2 \\
&&+\bigl [d_0+(1+d_1)B_1B_2\bigr ]\log (n_1n_2)
\end{eqnarray*}

is the usual BIC penalty with $d_0$ (null) and $d_1$ (alternative) density parameters. The first two terms in the BIC penalty penalizes the number of parameters in $\boldsymbol{\alpha }_r$, while the last term penalizes the number of parameters in $\boldsymbol{\Pi }, \nu _0$ and $\nu$. By Equation (1), the ICL criterion can also be written in terms of the observed data likelihood, the entropy of $\widehat{Q}$, and the BIC penalty. The entropy term favors compact, well-separated clusters and therefore guards against over-fitting.

## 4 Numerical experiments

We compared the proposed new procedure with the BH procedure, Storey’s $q$-value procedure, and the Sun and Cai procedure (Sun and Cai, 2007, SC). We implemented the new procedure with known null density and used the ICL criterion to perform model selection. For fair comparison, we also implemented the SC procedure with known null density.

We simulated data in the following scenarios. In all scenarios, undirected bipartite graphs of dimension $n_1=150$ by $n_2=200$ were generated and the standard normal distribution was chosen as the null.

- Data were sampled with $B_1 = B_2 = 3$ latent biclusters and equal group probabilities $\alpha _{q,1}=\alpha _{q,2} = 1/3$ for $q=1,2,3$. The connectivity parameters were set such that $\pi _{ql} = 0.1 \cdot {\bf 1}(q\ne l) + 0.8 \cdot {\bf 1}(q=l)$ for $q,l = 1,2,3$ where ${\bf 1}(\cdot )$ is the indicator function. Under the alternative we drew observations from $N(\mu _{ql},1)$. The three densest blocks are given a moderate signal, $\mu _{11}=\mu _{22}=\mu _{33}=1$, whereas all other blocks are given $\mu _{ql}=3$, ensuring that the sparser clusters have a larger effect size.

Besides the above modular bipartite graph, we also considered fixed graphs with other topological properties. Given $A$, data were sampled independently from $N(0,1)$ is $A_{ij}=0$ and from $N(2,1)$ if $A_{ij} = 1$.

**Figure 1:**
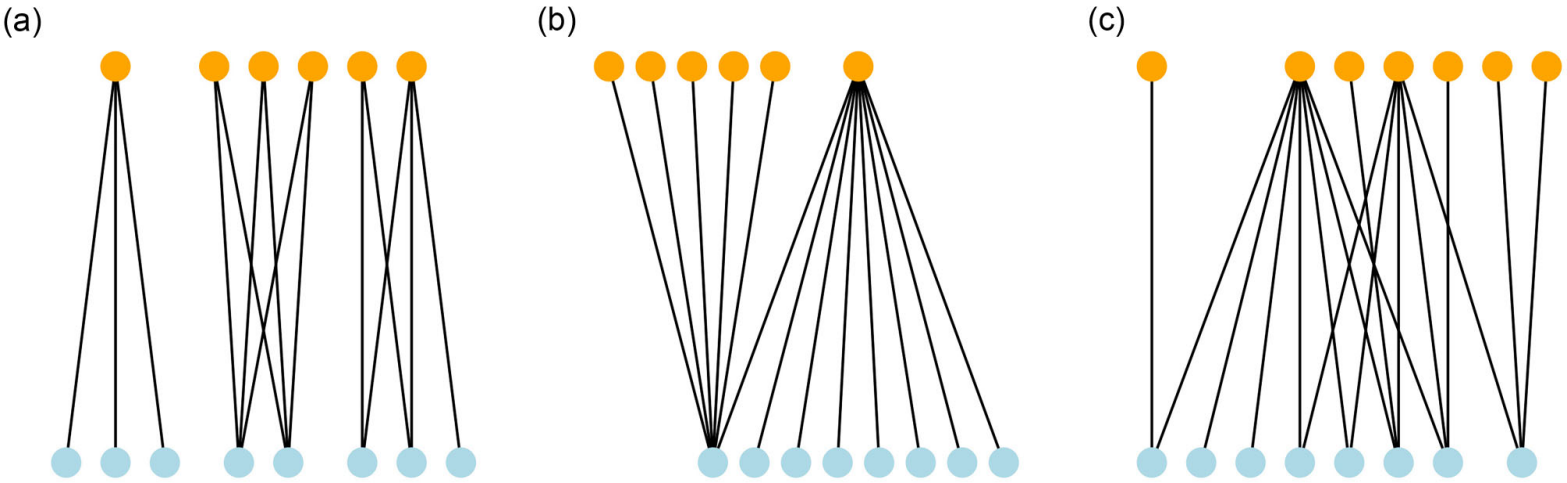
Illustrations of the latent bipartite network in the three scenarios: (a) three biclusters, (b) nested, and (c) preferential attachment. Color of the vertices indicates types of vertices in the graph.

- The latent graph $A$ is a fully nested bipartite graph (Figure 1 B) (Pavlopoulos et al., 2018). In other words, there is a single ‘generalist’ in each vertex type that is connected with all the nodes in the other vertex type.
- The latent graph $A$ is generated according to a bipartite preferential attachment model (Figure 1 C) (Guillaume and Latapy, 2006). At each step, a new type I vertex is added and its degree $d$ is sampled uniformly at random from the set $\lbrace 2,3,4,5,6\rbrace$. Then, for each of the $d$ edges of the new vertex, either a new type II vertex is added (with probability $1-\lambda$) or one is picked among the preexisting ones using preferential attachment (with probability $\lambda$). We set the parameter $\lambda =0.8$, which is the average ratio of preexisting type II vertices to which a new type I vertex is connected.

Figure 1 presents illustrations of the latent bipartite network in each scenario.

**Figure 2:**
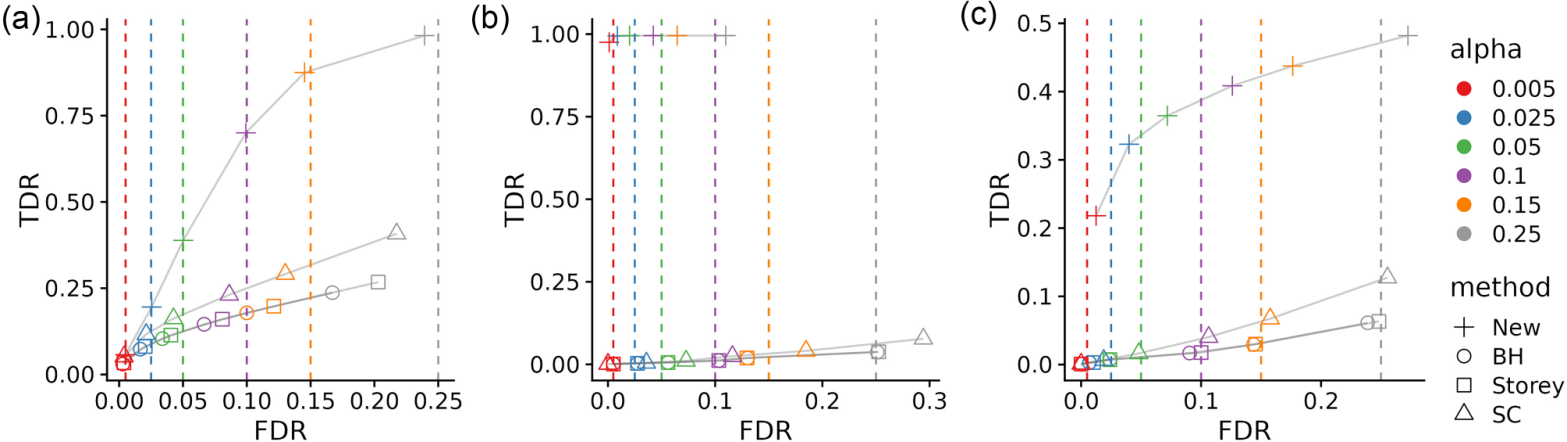
Plot of the empirical $({\rm FDR}, {\rm TDR})$ as functions of the nominal level $\alpha$ for the new procedure, BH, Storey’s $q$-value, and the SC procedure. Dashed lines indicate the nominal level $\alpha$.

We simulated 100 data sets for each scenario and applied every testing procedure at nominal FDR level $\alpha \in \lbrace 0.005,0.025, 0.05, 0.1, 0.15, 0.25\rbrace$. Figure 2 provides the ROC curves of the empirical FDR vs TDR as functions of $\alpha$.

Across all scenarios, the BH and Storey’s $q$-value procedures behave almost identically: they keep FDR near the nominal levels but achieve only modest TDR, with Storey’s method being slightly less conservative. The SC procedure controls the FDR at nominal levels in scenarios (a) and (c) but inflates FDR in scenario (b); its power is only marginally better than the $q$-value approach.

By contrast, the new procedure consistently outperforms existing methods across all scenarios. In scenario (a), its FDR stays close to the nominal level while its TDR is much higher. In 90% of the simulations the ICL criterion correctly selects three biclusters, and this accurate recovery of the latent structure markedly boosts the new method’s power. In scenario (b), although the data do not follow the assumed model, the ICL criterion chooses two biclusters in 83% of the simulations—one “generalist” cluster in each vertex set and a complementary “specialist” cluster. Estimated connection probabilities reflect the nested structure ($\approx 0.99$ for generalist–specialist edges, $\approx 0$ for specialist–specialist edges), enabling FDR below nominal levels and high TDR—something competing methods miss without explicit modeling. In scenario (c), the new method shows a slight FDR inflation, yet its ROC curve still dominates those of all other procedures, indicating robustness to model misspecification. In most simulations, the ICL criterion discovers three clusters in type-I vertices (high-, medium-, and low-degree nodes) and one in type-II vertices, yielding a meaningful partition and sustained power despite imperfect model fit.

In Web Appendix B.1, we demonstrate the new procedure is computationally more efficient and achieves improved FDR control than the original SBM. In Web Appendix B.2 & B.3, we provide additional simulation studies illustrating the performance of the new procedure when the number of clusters is specified differently from the truth or when the signal-to-noise ratio (SNR) is lower. The SNR in our model is determined jointly by the *block structure* and the *contrast between the null and alternative distributions*. If SNR is extremely low, parameter estimation may be inaccurate and the advantage of the new procedure over existing methods is likely to be small (Web Figure 3 and Web Table 1). Overall, by learning an appropriate biclustering, the new procedure maintains or improves FDR control and delivers substantially higher power, while existing methods remain conservative but underpowered.

## 5 Application to bacterial vaginosis

We analyzed paired microbiome–metabolome data from 131 Rwandan women reported in (McMillan et al., 2015). This study performed 16S rRNA sequencing to obtain taxonomic counts. After pre-processing (Web Appendix C.1), 49 taxa and 128 vaginal metabolites were retained. Among the 131 participants, 79 were classified as “normal”, 23 as BV, 22 as “intermediate” and 7 had no clinical diagnosis. Following McMillan et al. (2015), we pooled the BV and intermediate groups into a single disease cohort ($n=45$) and compared it with the normal cohort ($n=79$) to identify metabolic alterations associated with BV.

The proposed inference procedure requires a matrix ${\bf X}=(x_{ij})$ of $z$-scores, each summarizing the association between the $i$-th microbe and the $j$-th metabolite. Let ${\bf Y}_1\in \mathbb {R}^{m\times n_1}$ denote the microbiome data block and ${\bf Y}_2\in \mathbb {R}^{m\times n_2}$ the metabolome block with matched rows. The empirical Pearson correlation between the two blocks is $ {\widehat{\rho }}_{ij}=m^{-1}\sum _{k=1}^{m}{\widetilde{Y}}_{1,k,i}{\widetilde{Y}}_{2,k,j},$ with an asymptotic variance $ s_{ij}=m^{-1}\sum _{k=1}^{m} \bigl (2{\widetilde{Y}}_{1,k,i}{\widetilde{Y}}_{2,k,j}-{\widehat{\rho }}_{ij}{\widetilde{Y}}_{1,k,i} -{\widehat{\rho }}_{ij}{\widetilde{Y}}_{2,k,j}\bigr )^{2}$ (Cai and Liu, 2016), where ${\widetilde{{\bf Y}}}_k\ (k=1,2)$ denotes the standardized matrix after centering and scaling each column of ${\bf Y}_k$ to have mean zero and variance 1. To detect changes in microbe–-metabolite correlation between two clinical subgroups ($d=1,2$) with sample sizes $m_d$, let ${\widehat{\rho }}^{(d)}_{ij}$ and $s^{(d)}_{ij}$ be the within-group estimates defined above. The null hypothesis $H_{0,ij}:\rho ^{(1)}_{ij}=\rho ^{(2)}_{ij}$ is tested by $ x_{ij}={2\bigl ({\widehat{\rho }}^{(1)}_{ij}-{\widehat{\rho }}^{(2)}_{ij}\bigr )}/ {\sqrt{s^{(1)}_{ij}/m_1+s^{(2)}_{ij}/m_2}} .$

Dataset-specific diagnostics confirmed that the proposed inference procedure is appropriate for this dataset (Web Appendix C.3). Because the data were relatively noisy, we complemented the ICL criterion with cluster stability estimation (Web Appendix C.4 and Web Table 3) to avoid overfitting and identify the most reproducible block structure. The highest stability was achieved for $Q_1 = 4$ and $Q_2=1$, although its ICL value was slightly lower than the maximum value. We therefore applied the proposed testing procedure with $Q_1 = 4$ and $Q_2=1$, assuming the null density follows a standard normal distribution.

**Figure 3:**
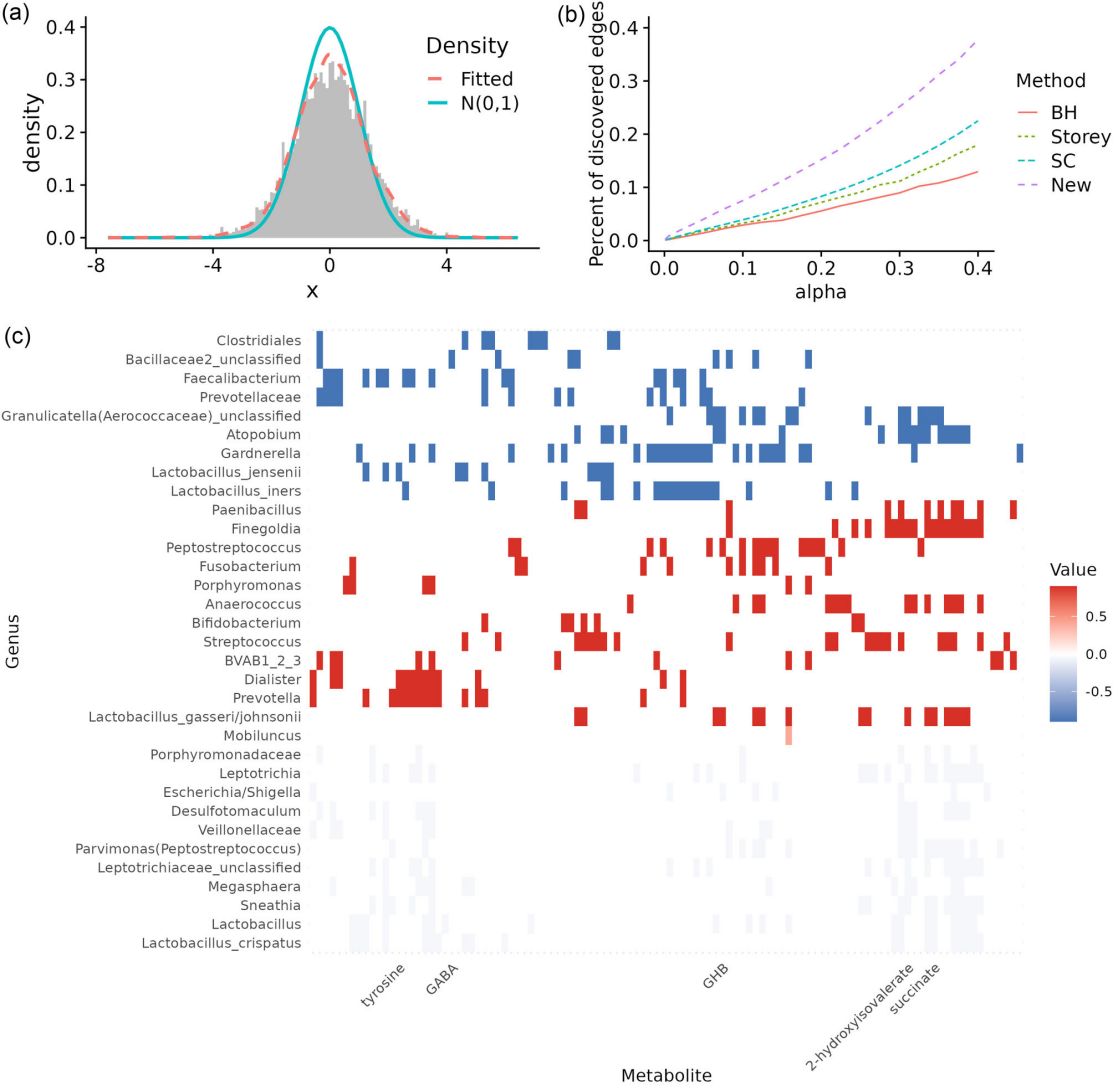
(A) Histogram of observed z-scores compared to the standard normal distribution and to the estimated marginal distribution by the proposed approach. (B) Percent of rejected edges as a function of the significance level $\alpha$ for the different procedures. (C) Heatmap of the inferred network at $\alpha =0.1$ with colors indicating the estimated mean values in each block. Labels of selected metabolites of interest to BV are shown.

Figure 3 A shows the distribution of the observed $z$-scores. Although the test statistics appear to be normally distributed, they deviate noticeably from the standard normal curve; the marginal density recovered by our method offers a better fit than the standard normal. Panel B demonstrates that, across every significance threshold, the new procedure yields more discoveries than competing methods, confirming its superior power. Not all discoveries identified by existing methods were declared significant by the new procedure (Web Figure 11). This is because, unlike existing approaches that evaluate each $z$-score independently, the proposed procedure pools information within blocks to make more informed decisions. By accounting for the global structure of the graph, the new procedure interprets some extreme test statistics as noise rather than true signals, and consequently finds no evidence of a difference between BV and healthy individuals for those associations.

Panel C displays the heatmap of the network inferred by our method at $\alpha =0.1$. Rows are ordered by the clusters identified by the algorithm, while columns are ordered by hierarchical clustering with the correlation distance. Taxa or metabolites with no detected associations to any other variables were removed. Each block is colored by its alternative mean values, indicating up-regulation (negative mean) or down-regulation (positive mean) in BV. The heatmap reveals a clear partition of taxa with distinct association patterns. Some taxa (e.g., *Lactobacillus gasseri/johnsonii, Bifidobacterium, Prevotella*) show stronger associations with metabolites in healthy individuals, whereas others (e.g., *L. iners, L. jensenii, Gardnerella, Atopobium*) exhibit stronger associations in BV. Although *Lactobacillus* and *L. crispatus* show differential associations with metabolites, the magnitude of these associations is small. Several taxa, including *Gardnerella, L. iners, L. jensenii, L. gasseri/johnsonii*, and *Finegoldia*, are connected to a large number of metabolites, suggesting that they may play central roles in microbial metabolic interactions during BV onset. A few key metabolites (e.g., *2-hydroxyisovalerate (2HV), succinate, GHB, GABA*, and *tyrosine*), previously identified as biomarkers for BV (McMillan et al., 2015; Srinivasan et al., 2015), were also highlighted in Figure 3 C. While McMillan et al. (2015) experimentally validated the production of *GHB* by *Gardnerella*, our differential network analysis suggests that the association between these two variables does not differ substantially between BV and healthy individuals. In contrast, *GHB* shows altered associations with *L. gasseri/johnsonii, Atopobium, Peptostreptococcus* and *Escherichia/Shigella*. Similarly, *2HV* displays altered associations with several taxa, including *L. gasseri/johnsonii, Atopobium, Sneathia*, and *Leptotrichia*. Although *succinate* was not significant in the univariate analysis of McMillan et al. (2015), our multivariate approach highlights its importance through “guilt by association”, consistent with previous reports implicating *succinate* in BV (Srinivasan et al., 2015). Together, these results indicate shifts in microbial metabolic interactions between BV and healthy states, and identify taxa–metabolite relationships that may warrant further investigation.

## 6 Discussion

Joint analysis of microbiome and metabolomic data can illuminate the metabolic circuitry that underlies host–microbe interactions and, ultimately, guide the design of microbiome-based therapeutics. This paper introduced a *bipartite noisy stochastic block model* that models the observed $z$-score matrix with a latent microbe–-metabolite interaction graph. By exploiting the latent block structure, the method (i) achieves reliable FDR control, (ii) delivers substantially higher power than existing procedures, and (iii) provides a biologically interpretable biclustering of taxa and metabolites. Simulations confirmed the gains in speed and accuracy over the original (noisy) SBM and over conventional FDR tools. The BV case study further demonstrated how the proposed testing procedure complements differential abundance analyses done in McMillan et al. (2015).

In our model, we can view the indicator matrix $A$ as an unobserved parameter drawn from a structured prior—in our case, a bipartite SBM. Estimating the hyper-parameters via marginal maximum likelihood, and replacing them in the $\ell$-value formula, is analogous to the empirical Bayes framework originally introduced in Efron et al. (2001). The key difference is that we encode *dependence* through the latent graph, so the procedure can borrow strength without assuming weak $p$-value correlations. Graphical models and network structures have been widely used to model dependence, including in the Bayesian literature. For instance, Peterson et al. (2015) use a Markov random field prior to encourage similarity among precision matrices across related groups, while Choi et al. (2025) introduce a hub random network prior to capture biologically realistic hub structures in directed graphs. These approaches and ours can be viewed as complementary perspectives on leveraging graphical dependence for high-dimensional inference.

Our method assumes each $x_{ij}$ has a tractable null and alternative density. In our simulations, the test statistics were drawn from Gaussian distributions. The Gaussian assumption is common in the Empirical Bayes and local false discovery rate literature (Efron et al., 2001; Efron, 2004; Sun and Cai, 2007; 2009; Efron, 2007). It is reasonable because many test statistics used in such procedures are, or can be transformed into, $z$-values. These include statistics from student’s $t$-test, $F$-test, $\chi ^2$ test, likelihood ratio test, Wald test and Score test. While it is possible to work directly with statistics that have heavier tails, transforming them to $z$-values simplifies the core analytical steps and provides robustness against nuisance parameters. If the test statistics instead follow a different parametric family that does not admit an easy transformation to $z$-values—for example, the exponential distribution—our framework can be adapted to estimate densities within that family (Web Appendix B.5). However, when the distributional family of the test statistics is completely unknown, we do not recommend using model-based FDR control methods, because these methods will fail to maintain valid FDR control.

The VEM algorithm can be viewed as an EM algorithm in which the E-step is approximated using variational inference techniques such as coordinate ascent variational inference (CAVI) (Blei et al., 2017). Although variational approximations are known to underestimate posterior variance (Blei et al., 2017), FDR in our framework depends primarily on posterior means rather than higher-order uncertainty. Consistent with this, our simulation studies show that the empirical FDR remains close to the nominal level across a wide range of scenarios, indicating that the variational approximation does not materially compromise inferential validity. The VEM algorithm scales linearly in the number of edges and converges rapidly. Under scenario (a) in Section 4, it took about 15 seconds for the algorithm to converge on a 24-in iMac with Apple M3 chip and 16 GB memory for a single pair of $(B_1,B_2)$. Yet the search over $(B_1,B_2)$ via the ICL criterion can still be time-consuming. A promising alternative is the greedy ICL maximization of (Kilian et al., 2024), which iteratively reassigns vertices and naturally prunes empty blocks.

Zero inflation is pervasive in microbiome data and poses challenges for reliable statistical inference. In addition, microbiome abundances are subject to total sum constraints. In the BV case study, we addressed these concerns using the mclr transformation, which preserves the original zero measurements and is overall rank-preserving. However, mclr-transformed components are still constrained to sum to zero. Our simulations applying the new procedure to simulated multi-omic profiles rather than test statistics demonstrate that both zero inflation and compositionality can affect FDR control, with the latter exerting a stronger influence (Web Appendix B.6; Web Figures 8 & 9). This observation underscores a more fundamental challenge that is not specific to the chosen test statistics: *testing correlations at the absolute abundance level is challenging when only relative abundances are observed*. To our knowledge, no established alternative currently provides a principled way to test correlations while simultaneously accounting for compositionality. Developing such correlation statistics remains an important but open methodological problem, which is beyond the scope of the present work. Practically speaking, if clr or mclr are employed to deal with the total sum constraint, care must be taken to interpret the results accordingly.

## Supplementary Material

ujag042_Supplemental_Files Web Appendices, Tables, Figures from additional simulation and case studies, and data and code for reproducing the results referenced in Sections 3–6 are available with this article at the Biometrics website on Oxford Academic.

## Data Availability

Data used in this paper to support our findings are publicly available. See Supplementary Material for details.
