# Supplementary material for: Inference for microbe–metabolite association networks using a latent graph model
Source: Biometrics. 2026 Mar 10;82(1):ujag042. doi: 10.1093/biomtc/ujag042 (PMC13017156; doi:10.1093/biomtc/ujag042)
Supplement: ujag042_Supplemental_Files — Web Appendices, Tables, Figures from additional simulation and case studies, and data and code for reproducing the results referenced in Sections 3–6 are available with this article at the Biometrics website on Oxford Academic. [file ujag042_supplemental_files.zip › biometric_supp.pdf]

Supplementary Material for "Inference for microbe–metabolite association networks using a latent graph model" by Jing Ma

Web Appendix A Details of the Variational E-step Derivation

In the variational E-step, we need to maximize the ELBO to solve for the variational parameters $\boldsymbol{\beta}_1$ and $\boldsymbol{\beta}_2$.

Denote by $\mathcal{A} = \{(i,j) : 1 \le i \le n_1, 1 \le j \le n_2\}$ the set of all possible pairs that we wish to test. The complete-data likelihood can be rewritten as

$$
\begin{aligned}
L(\mathbf{X}, \mathbf{A}, \mathbf{Z}_1, \mathbf{Z}_2; \boldsymbol{\theta}) = & \prod_{\substack{(i,j)\in\mathcal{A}: \\ A_{ij}=0}} g_{\nu_0}(x_{ij}) \prod_{q=1}^{B_1}\prod_{l=1}^{B_2} \prod_{\substack{(i,j):A_{ij}=1 \\ Z_{i,1}=q, Z_{j,2}=l}} g_{\nu_{ql}}(x_{ij}) \times \\
& \prod_{q=1}^{B_1}\prod_{l=1}^{B_2} \pi_{ql}^{M_{ql}} (1-\pi_{ql})^{\widetilde{M}_{ql}} \times \prod_{r=1}^{2}\prod_{q=1}^{B_r} \alpha_{q,r}^{\sum_{i=1}^{n_r} \mathbf{1}(Z_{i,r}=q)},
\end{aligned}
$$

where

$$
\begin{aligned}
M_{ql} &= \#\{(i,j) \in \mathcal{A} : A_{ij} = 1, Z_{i,1} = q, Z_{j,2} = l\}, \\
\widetilde{M}_{ql} &= \#\{(i,j) \in \mathcal{A} : A_{ij} = 0, Z_{i,1} = q, Z_{j,2} = l\}.
\end{aligned}
$$

Let $\widetilde{E}_{\boldsymbol{\beta}_1,\boldsymbol{\beta}_2}$ and $\widetilde{P}_{\boldsymbol{\beta}_1,\boldsymbol{\beta}_2}$ denote, respectively, the expected value and probability distribution function with respect to the variational distribution. Substituting Q with the factorized variational distribution, we get

$$
\begin{aligned}
\widetilde{E}_{\boldsymbol{\beta}_1,\boldsymbol{\beta}_2}[\log L(\mathbf{X}, \mathbf{A}, \mathbf{Z}_1, \mathbf{Z}_2; \boldsymbol{\theta})] = & \sum_{(i,j)\in\mathcal{A}} \widetilde{P}_{\boldsymbol{\beta}_1,\boldsymbol{\beta}_2}(A_{ij} = 0) \log g_{0,\nu_0}(x_{ij}) \\
& + \sum_{q=1}^{B_1}\sum_{l=1}^{B_2}\sum_{(i,j)\in\mathcal{A}} \widetilde{P}_{\boldsymbol{\beta}_1,\boldsymbol{\beta}_2}(A_{ij} = 1, Z_{i,1} = q, Z_{j,2} = l) \log g_{\nu_{ql}}(x_{ij}) \\
& + \sum_{q=1}^{B_1}\sum_{l=1}^{B_2}\sum_{(i,j)\in\mathcal{A}} \widetilde{P}_{\boldsymbol{\beta}_1,\boldsymbol{\beta}_2}(A_{ij} = 1, Z_{i,1} = q, Z_{j,2} = l) \log \pi_{ql} \\
& + \sum_{q=1}^{B_1}\sum_{l=1}^{B_2}\sum_{(i,j)\in\mathcal{A}} \widetilde{P}_{\boldsymbol{\beta}_1,\boldsymbol{\beta}_2}(A_{ij} = 0, Z_{i,1} = q, Z_{j,2} = l) \log(1-\pi_{ql}) \\
& + \sum_{r=1}^{2}\sum_{q=1}^{B_r}\sum_{i=1}^{n_r} \widetilde{E}_{\boldsymbol{\beta}_1,\boldsymbol{\beta}_2}[\mathbf{1}(Z_{i,r} = q)] \log \alpha_{q,r}
\end{aligned}
$$

To calculate the entropy of the factorized variational distribution Q, note that

$$\widetilde{E}_{\beta_1,\beta_2}[\log Q] = \sum_{q=1}^{B_1}\sum_{l=1}^{B_2}\sum_{(i,j)\in\mathcal{A}} \widetilde{P}_{\beta_1,\beta_2}(A_{ij}=1, Z_{i,1}=q, Z_{j,2}=l)\log\rho_{ij}^{ql} + \sum_{q=1}^{B_1}\sum_{l=1}^{B_2}\sum_{(i,j)\in\mathcal{A}} \widetilde{P}_{\beta_1,\beta_2}(A_{ij}=0, Z_{i,1}=q, Z_{j,2}=l)\log(1-\rho_{ij}^{ql}).$$

And

$$\widetilde{E}_{\beta_1,\beta_2}[\log(\prod_{i=1}^{n_1}\beta_{i,Z_{i,1},1})] = \sum_{q=1}^{B_1}\sum_{i=1}^{n_1}\widetilde{E}_{\beta_1,\beta_2}[\mathbf{1}(Z_{i,r}=q)]\log\beta_{i,q,1}.$$

By Bayes rule, the conditional distribution of A given block memberships Z_1 and Z_2 follows a Bernoulli distribution with conditional probability

$$P(A_{ij}=1 \mid Z_{i,1}=q, Z_{j,2}=l, \mathbf{X}) = \frac{\pi_{ql}g_{\nu_{ql}}(x_{ij})}{\pi_{ql}g_{\nu_{ql}}(x_{ij}) + (1-\pi_{ql})g_{\nu_0}(x_{ij})} := \rho_{ij}^{ql}.$$

Putting all the pieces together, we obtain

$$\begin{aligned}\mathrm{ELBO} = &-\sum_{q=1}^{B_1}\sum_{l=1}^{B_2}\sum_{(i,j)\in\mathcal{A}} \beta_{i,q,1}\beta_{j,l,2}\left\{\rho_{ij}^{ql}\log\rho_{ij}^{ql} + (1-\rho_{ij}^{ql})\log(1-\rho_{ij}^{ql})\right\} + \\ &\sum_{q=1}^{B_1}\sum_{l=1}^{B_2}\sum_{(i,j)\in\mathcal{A}} \beta_{i,q,1}\beta_{j,l,2}\left\{\rho_{ij}^{ql}\log\pi_{ql} + (1-\rho_{ij}^{ql})\log(1-\pi_{ql})\right\} + \\ &\sum_{(i,j)\in\mathcal{A}}\log g_{0,\nu_0}(x_{ij})\sum_{q=1}^{B_1}\sum_{l=1}^{B_2}\beta_{i,q,1}\beta_{j,l,2}(1-\rho_{ij}^{ql}) + \\ &\sum_{q=1}^{B_1}\sum_{l=1}^{B_2}\sum_{(i,j)\in\mathcal{A}}\rho_{ij}^{ql}\beta_{i,q,1}\beta_{j,l,2}\log g_{\nu_{ql}}(x_{ij}) + \\ &\sum_{q=1}^{B_1}\sum_{i=1}^{n_1}\beta_{i,q,1}\log\frac{\alpha_{q,1}}{\beta_{i,q,1}} + \sum_{l=1}^{B_2}\sum_{j=1}^{n_2}\beta_{i,l,2}\log\frac{\alpha_{l,2}}{\beta_{j,l,2}}.\end{aligned}$$

After rearranging terms, we can rewrite the ELBO as

$$\mathrm{ELBO} = \sum_{q=1}^{B_1}\sum_{i=1}^{n_1}\beta_{i,q,1}\log\frac{\alpha_{q,1}}{\beta_{i,q,1}} + \sum_{l=1}^{B_2}\sum_{j=1}^{n_2}\beta_{i,l,2}\log\frac{\alpha_{l,2}}{\beta_{j,l,2}} + \sum_{q=1}^{B_1}\sum_{l=1}^{B_2}\sum_{(i,j)\in\mathcal{A}}\beta_{i,q,1}\beta_{j,l,2}d_{ij}^{ql}.$$

The partial derivative of the ELBO with respect to $\beta_{i,q,r}$ is given by

$$\begin{aligned}\frac{\partial}{\partial\beta_{i,q,1}}\mathrm{ELBO} &= \log\alpha_{q,1} - \log\beta_{i,q,1} - 1 + \sum_{l=1}^{B_2}\sum_{j=1}^{n_2}\beta_{j,l,2}d_{ij}^{ql}, \\ \frac{\partial}{\partial\beta_{j,l,2}}\mathrm{ELBO} &= \log\alpha_{q,2} - \log\beta_{j,l,2} - 1 + \sum_{q=1}^{B_1}\sum_{i=1}^{n_1}\beta_{i,q,1}d_{ij}^{ql}.\end{aligned}$$

Scenario the partial derivative to zero yields

$$\beta_{i,q,1} = \alpha_{q,1} \exp\left(\sum_{l=1}^{B_2}\sum_{j=1}^{n_2} \beta_{j,l,2} d_{ij}^{ql} - 1\right), \quad \beta_{j,l,2} = \alpha_{l,2} \exp\left(\sum_{q=1}^{B_1}\sum_{i=1}^{n_1} \beta_{i,q,1} d_{ij}^{ql} - 1\right). \tag{1}$$

The probabilities also satisfy the constraint $\sum_{q=1}^{B_r} \beta_{i,q,r} = 1$ for all i and r. There are no explicit solutions to these equations (1), but they can be achieved via fixed-point iterations.

Web Appendix B Additional Simulation Studies

We performed additional simulations to evaluate the performance of the new procedure. We also added settings where we began generation of microbiome and metabolomic profiles to evaluate how zero inflation affects the new procedure.

Web Appendix B.1 Comparison to noisySBM

We sampled a bipartite network $\mathbf{A}$ of size $n_1 = 40$ and $n_2 = 60$ with 2 row clusters and 3 column clusters. Nodes were assigned equal group probabilities among the rows and columns. The connectivity parameters were set such that $\pi_{1,1} = \pi_{2,2} = 0.8$ and 0.1 elsewhere. Given $\mathbf{A}$, observed data $x_{i,j}$ was sampled independently from a standard normal distribution if $A_{i,j} = 0$ and from $\mathcal{N}(\mu, 0.25)$ if $A_{i,j} = 1$. The matrix $\mathbf{X}$ was used as input to the new procedure. The input to noisySBM was $\widetilde{X} = \begin{pmatrix} \mathbf{X}_0 & \mathbf{X} \\ \mathbf{X}' & \mathbf{X}_1 \end{pmatrix}$, where entries in $\mathbf{X}_0$ and $\mathbf{X}_1$ were sampled independently from a standard normal distribution. This ensures that the latent graph $\widetilde{\mathbf{A}}$ given $\widetilde{\mathbf{X}}$ corresponds to the same bipartite network $\mathbf{A}$.

We implemented both the bipartite and original noisySBM (Rebafka et al., 2022) under the ideal scenario that the null density and the true number of clusters are both known. Web Figure 1 compares their empirical FDR, TDR, and run time across different values of μ. At $\mu = 1$, noisySBM shows severely inflated FDR and a slightly lower TDR than the new method. This is because noisySBM produces poor estimation of latent block memberships which compromises its performance on hypothesis testing. The new procedure, while clustering rows imperfectly, achieves better column clustering and therefore a smaller FDR. As the signal increases, the new method's FDR approaches the nominal level, whereas noisySBM's FDR first falls and then rises because it increasingly misclassifies edges between nodes of the same type. TDRs are nearly identical for both methods across most settings. Computationally, however, the new procedure is at least 20 times faster: it solves two smaller problems, whereas noisySBM must infer from the data that cross-type connections are absent—a task that markedly slows its inference. Similar advantages of the bipartite SBM formulation—in both speed and accuracy of community detection—have also been reported when the observation is noiseless; that is when the network $\mathbf{A}$ is observed (Larremore et al., 2014).

Web Appendix B.2 Sensitivity to the number of clusters

To further assess robustness, we re-ran scenario (a) from the main manuscript with $B_1 = B_2 = 1$ and $B_1 = B_2 = 5$, while the true number of clusters was $B_1 = B_2 = 3$. The results

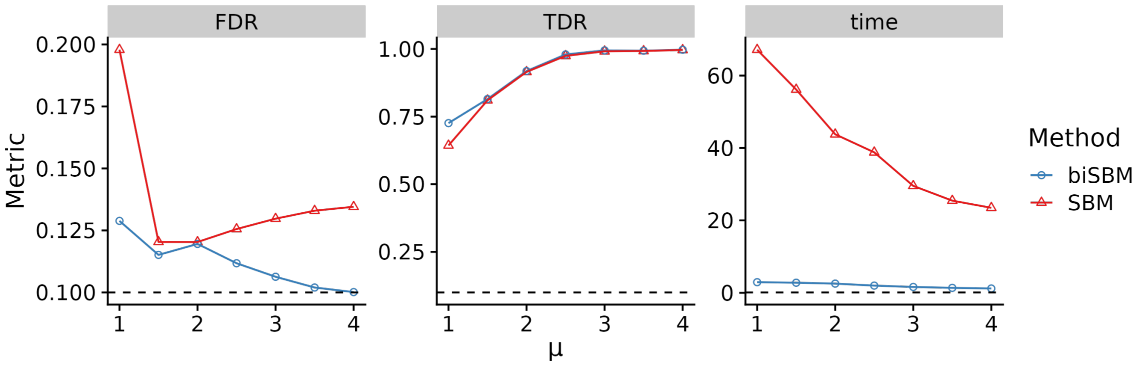

Web Figure 1: Performance of noisySBM versus its bipartite formulation in multiple testing, evaluated as a function of the alternative mean. Dashed line indicates the nominal level 0.1.

in Web Figure 2 show that the ROC curve of the new procedure coincides with that of the Sun & Cai procedure (Sun and Cai, 2007) when $B_1 = B_2 = 1$ and surpasses the Sun & Cai procedure when $B_1 = B_2 = 5$ or when the clusters are estimated by ICL. This analysis demonstrates that the proposed procedure remains robust and maintains power advantages under moderate deviations from the true cluster structure.

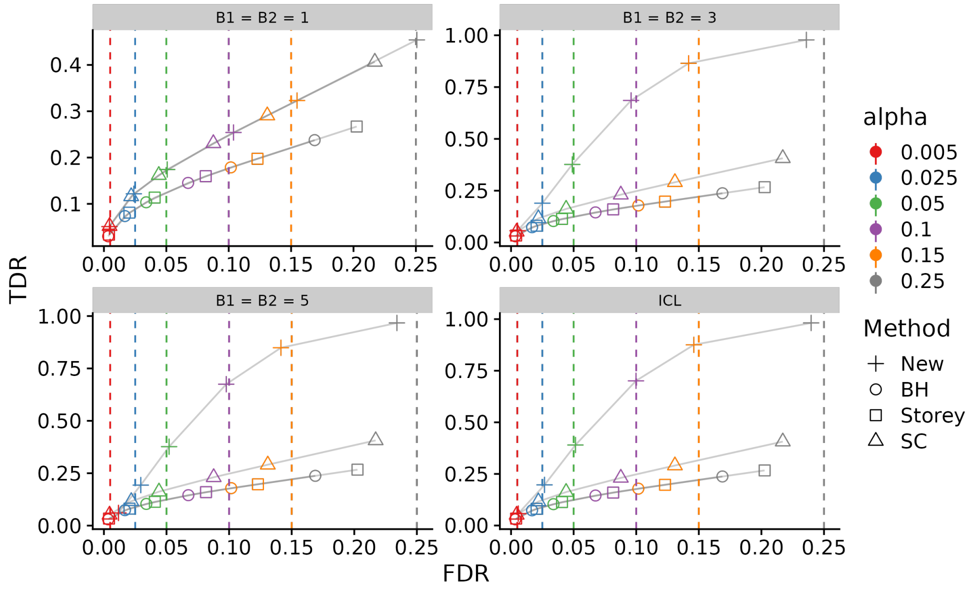

Web Figure 2: Impact of the number of clusters on FDR control. The true number of clusters is $B_1 = B_2 = 3$.

Web Appendix B.3 Scenarios with lower signal-to-noise ratios

The signal-to-noise ratio (SNR) in our model is determined jointly by the *block structure* and the *contrast between the null and alternative distributions*. In the bipartite SBM, we control the block structure through the within-block and between-block edge probabilities $(p_{\text{within}}, p_{\text{btw}})$, setting $p_{\text{btw}} < p_{\text{within}}$ so that edges are more likely to occur within blocks. To control the difference between the null and alternative distributions, we fix the Gaussian variance to 1, assign the null mean to 0, and set the alternative means to μ_{within} for non-nulls within blocks and μ_{btw} for non-nulls between blocks, with $\mu_{\text{within}} \leq \mu_{\text{btw}}$ since between-block edges are sparser.

As illustrated in Web Figure 3, when p_{btw} and p_{within} are close, the block structure is very weak. The new procedure and the SC method—both more powerful than BH and Storey's q-value procedure—yield nearly identical results, which is expected since the new method effectively estimates a single block under weak structure. When the block structure becomes stronger ($p_{\text{within}} = 0.5, 0.8$) but the mean difference remains moderate, the two methods still perform comparably, with the new procedure showing a slight power advantage in some scenarios. The new procedure achieves the largest power gains when both the block structure and the mean difference are strong. Across all scenarios, it maintains valid FDR control, even when the block structure is not accurately recovered at lower SNR levels (see Web Table 1).

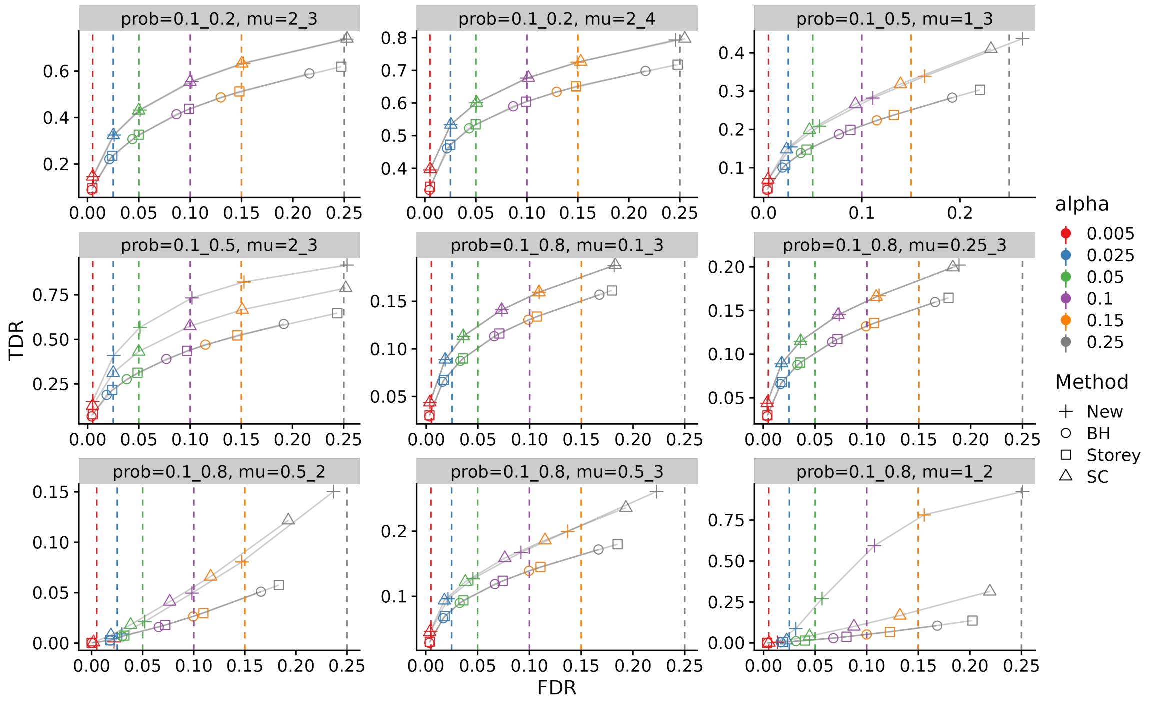

Web Figure 3: Performance of the new procedure compared to existing methods at varying SNR. In all scenarios, the ICL criterion was used to determine the number of clusters. The title of each panel indicates $(p_{\text{btw}}, p_{\text{within}}, \mu_{\text{within}}, \mu_{\text{btw}})$. All other data generating parameters were kept identical to those in Scenario (a).

The accuracy of the ICL criterion in recovering the true number of clusters depends strongly on the signal-to-noise ratio (SNR). As shown in Web Table 1, the estimated number of clusters approaches the true value when the SNR is high, but tends to collapse toward one cluster under low SNR scenarios. The seemingly higher adjusted Rand index observed at low SNR arises because most nodes are assigned to the same cluster.

Web Table 1: Adjusted Rand indices for row and column clustering, and estimated numbers of clusters under varying signal-to-noise ratio (SNR) settings when test statistics were generated from Gaussian distributions. The two probabilities represent the between- and within-cluster edge probabilities $(p_{\text{btw}}, p_{\text{within}})$, while the two mean parameters denote the within- and between-cluster means $(\mu_{\text{within}}, \mu_{\text{btw}})$ of the alternative distributions. In all scenarios, the ICL criterion was used to determine the number of clusters.

	Scenario	ARI_row	ARI_col	mean(Q)	sd(Q)
1	prob=0.1_0.2, mu=2_3	0.80	0.86	1.83	1.26
2	prob=0.1_0.2, mu=2_4	0.99	0.99	1.04	0.32
3	prob=0.1_0.5, mu=1_3	0.45	0.48	2.65	1.47
4	prob=0.1_0.5, mu=2_3	0.98	0.98	3.19	0.51
5	prob=0.1_0.8, mu=0.1_3	0.93	0.93	1.29	0.76
6	prob=0.1_0.8, mu=0.25_3	0.92	0.96	1.33	0.87
7	prob=0.1_0.8, mu=0.5_2	0.31	0.37	3.56	1.17
8	prob=0.1_0.8, mu=0.5_3	0.61	0.61	2.24	1.45
9	prob=0.1_0.8, mu=1_2	0.95	0.95	3.72	0.84

Web Appendix B.4 Comparison of shared discoveries across methods

Web Figure 4 shows the proportion of shared discoveries between each method and the proposed procedure across varying FDR thresholds and varying SNR. The denominator is the number of discoveries identified by each method. Not all discoveries identified by existing methods were declared significant by the proposed procedure. This is because, unlike existing approaches that evaluate each z-score independently, the proposed procedure pools information within blocks to make more informed decisions. By accounting for the global structure of the graph, the new procedure interprets some extreme test statistics as noise rather than true signals, and consequently finds no evidence of an association.

Web Appendix B.5 Scenarios with Gamma-distributed statistics

For sensitivity analysis, we explored test statistics drawn from Gamma distributions, which exhibit heavier tails than the Gaussian. The chi-squared distribution commonly used in likelihood ratio tests is a special case of the Gamma family, with rate parameter $\lambda = 0.5$. Under the null hypothesis, the test statistics follow a Gamma$(1, 1)$ distribution—equivalently, an Exp(1) distribution—whereas under the alternative they follow Gamma(α, λ) with shape parameter α and rate parameter λ. All other data generating parameters were kept identical to those in Scenario (a). Web Figure 5 compares the performance of the proposed method with the BH and Storey's q-value procedures when the block structure is strong. The SC

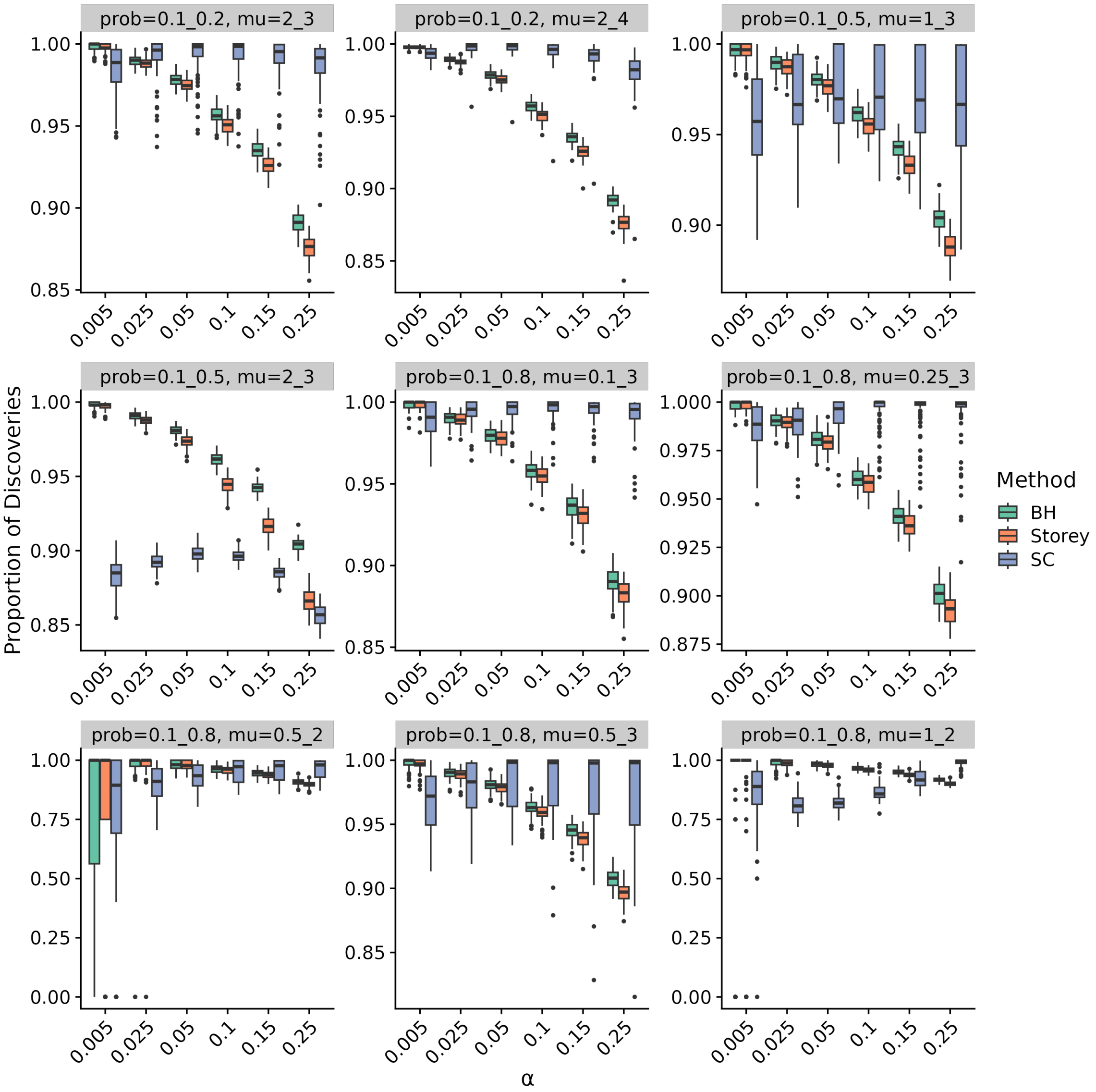

Web Figure 4: Proportion of shared discoveries between each method and the proposed procedure across varying FDR thresholds and varying SNR. The denominator is the number of discoveries identified by each method. Larger values indicate that more discoveries identified by existing methods were also declared significant by the new procedure.

method was excluded because it requires z-values, which are not available in this setting. The proposed procedure exhibits higher power than existing approaches in some scenarios, although it can also underperform in others. When both the null and alternative distributions are exponential (i.e., Gamma with shape parameter equal to 1), the proposed method achieves a noticeable power gain relative to existing procedures albeit with slight FDR inflation. In contrast, when the alternative follows a more general Gamma distribution, the improvement is more modest and the inflation in FDR is more severe. Moreover, selecting

the number of clusters using the ICL criterion generally enhances clustering accuracy, as quantified by the adjusted Rand index (see Web Table 2), though this does not necessarily lead to better FDR control. This occurs because ICL focuses on maximizing the goodness of fit of the block structure to the data, but parameter estimation in Gamma mixtures is substantially more challenging than in Gaussian mixtures, due to their heavier tails and asymmetry. When the block structures are weaker (Web Figure 6), the new procedure does not have advantage over existing methods.

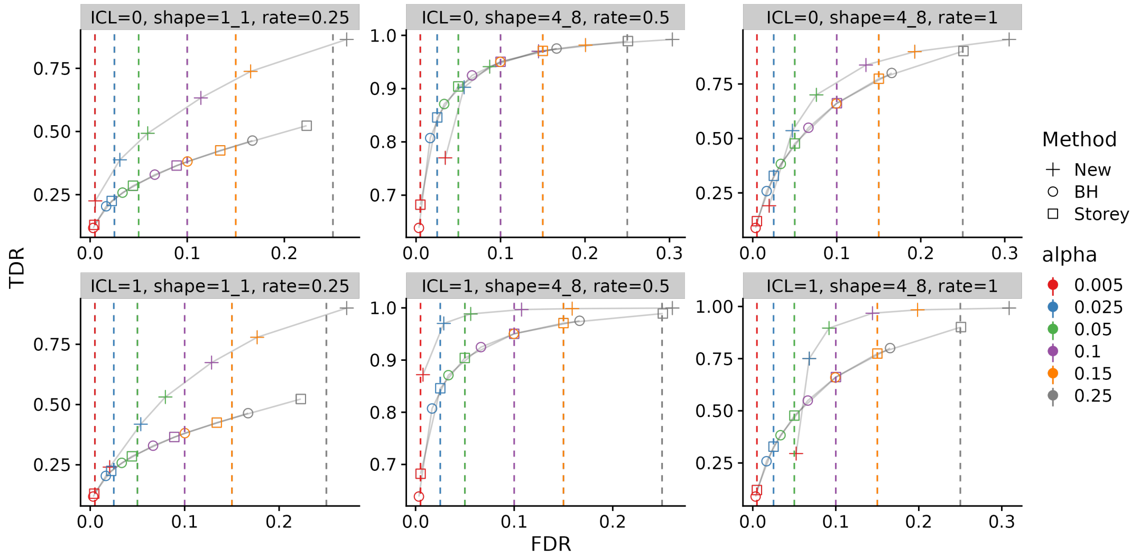

Web Figure 5: Performance of the new procedure compared to existing methods when test statistics are generated from Gamma distributions and block structures are strong ($p_{\text{btw}} = 0.1, p_{\text{within}} = 0.8$). The title of each panel indicates whether ICL was used to select the number of clusters (ICL=1), the shape parameters α_{within} and α_{btw}, and the rate parameter λ. All other data generating parameters were kept identical to those in Scenario (a).

Overall, these findings suggest that the advantage of the new procedure is modest when the test statistics arise from heavier-tailed distributions. In these cases, p-value based procedures such as Storey's q is recommended instead.

Web Appendix B.6 Impact of zero-inflation

We began with the generation of microbiome and metabolomic profiles to evaluate how zero inflation affects the performance of the proposed testing procedure. Specifically, we simulated two samples with correlation matrices $\mathbf{\Sigma}_1$ and $\mathbf{\Sigma}_2$, where the off-diagonal block of the differential network $\mathbf{\Sigma}_1 - \mathbf{\Sigma}_2$ followed a stochastic block model (SBM) structure (Web Figure 7). To introduce heteroscedasticity, we scaled the correlation matrices by standard deviations uniformly sampled from $[1, 3]$. Data in each sample were then drawn from a multivariate normal distribution with means uniformly sampled from $[\mu, 4]$. Microbiome counts were obtained by exponentiating and rounding the simulated abundances to the

Web Table 2: Adjusted Rand indices for row and column clustering under different settings when test statistics were drawn from Gamma distributions and block structures are strong ($p_{\text{btw}} = 0.1, p_{\text{within}} = 0.8$). The two shape parameters represent the within- and between-cluster shapes of the alternative distribution, and the rate parameter under the null is 1 in all scenarios. ICL=0 indicates the number of clusters was pre-specified as $B_1 = B_2 = 3$, while ICL=1 means that the number of clusters was selected by the ICL criterion.

	Scenarios	ARI_row	ARI_col	mean(Q)	sd(Q)
1	ICL=0, shape=1_1, rate=0.25	0.91	0.92	3.00	0.00
2	ICL=0, shape=4_8, rate=0.5	0.88	0.87	3.00	0.00
3	ICL=0, shape=4_8, rate=1	0.81	0.83	3.00	0.00
4	ICL=1, shape=1_1, rate=0.25	0.98	0.99	3.31	0.60
5	ICL=1, shape=4_8, rate=0.5	0.98	0.98	4.08	0.93
6	ICL=1, shape=4_8, rate=1	0.96	0.94	4.00	0.91

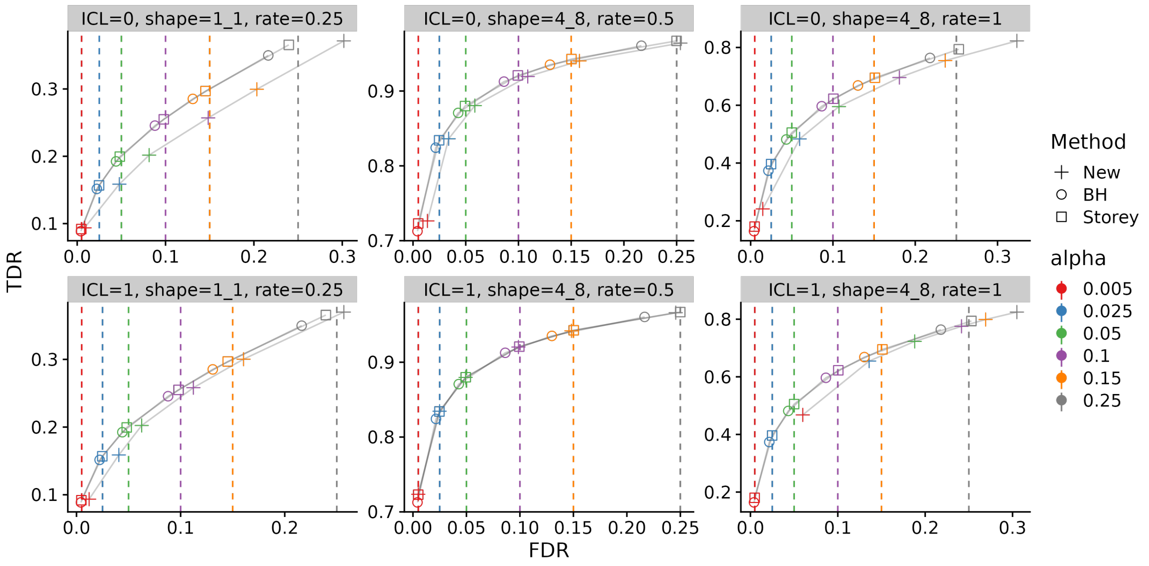

Web Figure 6: Performance of the new procedure compared to existing methods when test statistics are generated from Gamma distributions and block structures are weak ($p_{\text{btw}} = 0.1, p_{\text{within}} = 0.2$). The title of each panel indicates whether ICL was used to select the number of clusters (ICL=1), the shape parameters α_{within} and α_{btw}, and the rate parameter λ. All other data generating parameters were kept identical to those in Scenario (a).

nearest integers. The lower bound μ controls the degree of zero inflation. Smaller μ values produced higher proportions of zeros (approximately 10% when $\mu = 0$ and 37% when $\mu = 4$ at sample size 400).

We fixed the number of clusters at the true value and examined performance as a function of both zero inflation (via μ) and sample size. Web Figure 8 displays ROC curves for each method at sample size $m = 400$. Because the magnitude of correlations in the differential

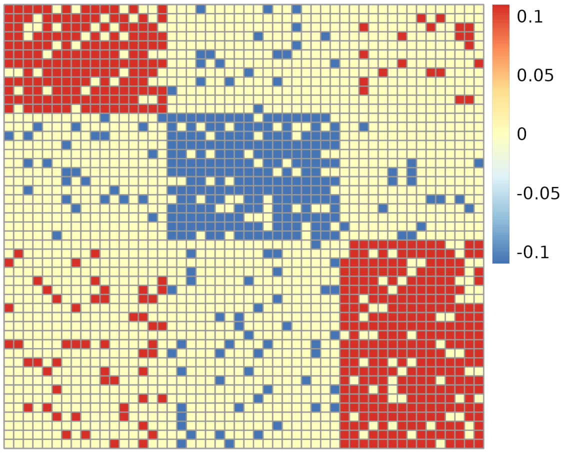

Web Figure 7: The off-diagonal block in the differential network $\mathbf{\Sigma}_1 - \mathbf{\Sigma}_2$.

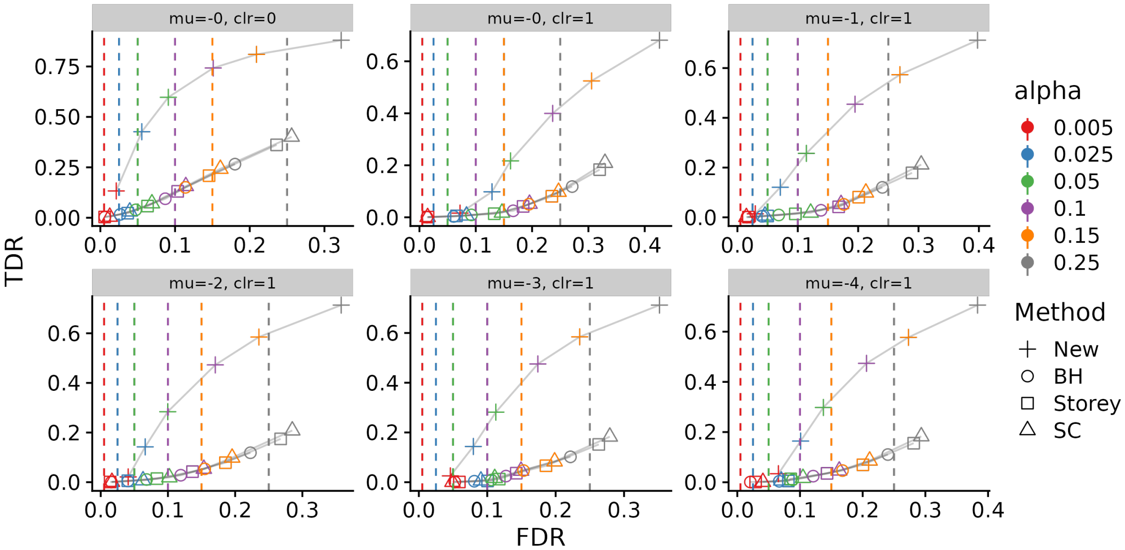

Web Figure 8: Impact of zero inflation on FDR control when generating microbiome and metabolomic data from pre-specified covariance matrices. Here the sample size is $m = 400$, the number of taxa is $n_1 = 49$ and the number of metabolites is $n_2 = 50$. The differential cross-correlation network has $B_1 = B_2 = 3$ biclusters. Within each panel, clr=0 indicates analysis done using the latent Gaussin data, while clr=1 indicates using observed data after applying the mCLR transformation.

network is modest (0.11), relatively large samples are required to detect differences. Under this setting, the proposed procedure applied to the latent Gaussian data shows moderate inflation of FDR but still achieves superior ROC performance relative to existing methods, possibly due to additional variability introduced in estimating the test statistics. When applied to mclr-transformed relative abundance data, all methods except the BH procedure

exhibit FDR inflation. The severity of FDR inflation first decreases and then increases with increasing zero inflation. At a smaller sample size ($m = 200$; Web Figure 9), the performance of all methods deteriorates.

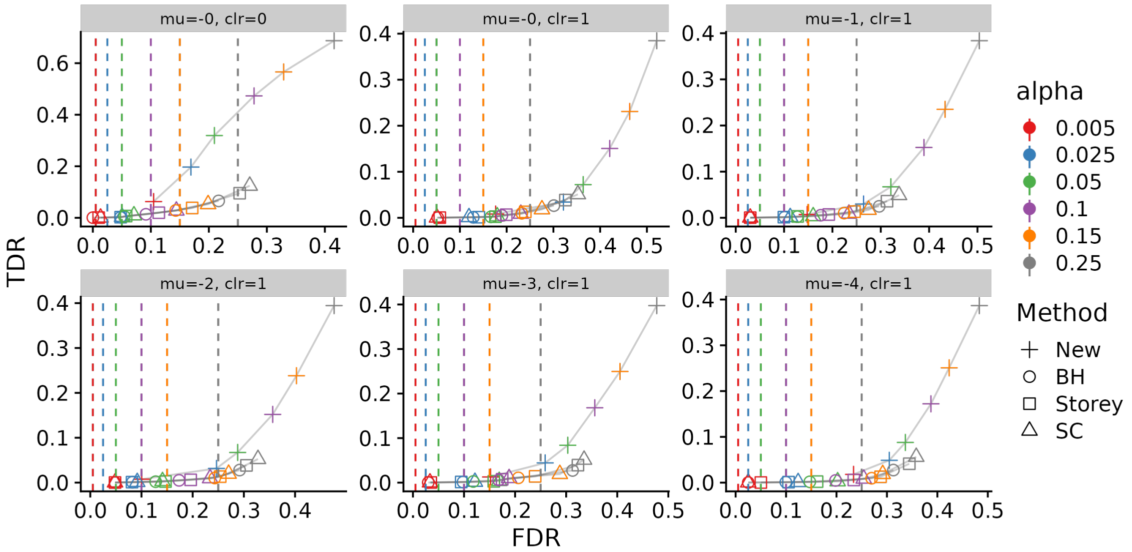

Web Figure 9: Impact of zero inflation on FDR control when generating microbiome and metabolomic data from pre-specified covariance matrices. Here the sample size is $m = 200$, the number of taxa is $n_1 = 49$ and the number of metabolites is $n_2 = 50$. The differential cross-correlation network has $B_1 = B_2 = 3$ biclusters. Within each panel, clr=0 indicates analysis done using the latent Gaussin data, while clr=1 indicates using observed data after applying the mCLR transformation.

Taken together, these results demonstrate that zero inflation can distort the behavior of the asymptotic test statistics and affect FDR control. However, the compositional constraint appears to exert a stronger influence, as evidenced by the comparison between panels ($\mu = 0, \text{clr} = 0$) and ($\mu = 0, \text{clr} = 1$) in Web Figures 8 and 9, because the mclr-transformed components are still constrained to sum to zero. This observation underscores a more fundamental challenge that is not specific to the chosen test statistics: testing correlations at the absolute abundance level is challenging when only relative abundances are observed. To our knowledge, no established alternative currently provides a principled way to test correlations while simultaneously accounting for compositionality. Developing such correlation statistics remains an important but open methodological problem, which is beyond the scope of the present work.

Practically speaking, if clr or mclr are employed to deal with the total sum constraint, then care must be taken to interpret the results accordingly.

Web Appendix C   Additional Details of the BV Case Study

Web Appendix C.1   Data preprocessing

Operational taxonomic units (OTUs) were clustered at 97% sequence identity, and taxonomic assignments were based on best hits from the Ribosomal Database Project (Cole et al., 2014), with manual curation against the Greengenes (DeSantis et al., 2006) and an in-house vaginal sequence database. Taxa showing at least 95% sequence similarity were retained, OTUs were aggregated to the genus level (except for Lactobacillus), and rare OTUs (less than 0.5% in any sample) were removed. A no-template control was included to identify and control for background contamination. After these quality filters, 51 bacterial taxa were retained. We removed one taxon present in only 13 participants and merged duplicate *Bacteroides* counts, leaving 49 distinct taxa. The proportion of zeros is about 11% and only 7 taxa have more than 30% of zeros. Web Figure 10 shows the proportion of zeros per taxon. To correct for varying sequencing depth, we applied the modified centered log-ratio (mclr) transform, which rescales positive counts while leaving zeros unchanged (Ma, 2021; Yoon et al., 2019). Gas-chromatography mass spectrometry was used to obtain vaginal metabolomic data. Chromatogram files were deconvoluted using AMDIS (Stein, 1999) (high resolution, medium sensitivity) and aligned in SpectConnect (Styczynski et al., 2007) with a low support threshold. Metabolites detected in blank swabs or identified as derivatization artifacts were removed. Intensities were normalized to ribitol and log-transformed using the additive log-ratio (alr) transformation (Aitchison and Bacon-Shone, 1984) after imputing zeros as two-thirds of the minimum detected value per metabolite. Metabolite identities were assigned using the NIST 11 library and confirmed with authentic standards when available.

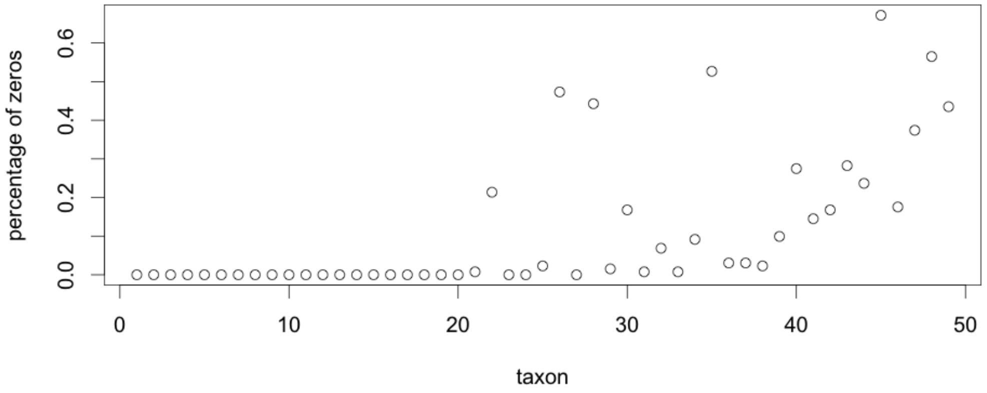

Web Figure 10: Percentage of zeros per taxon in the BV data set.

Web Appendix C.2 Comparison of shared discoveries across methods

Web Figure 11 shows the proportion of shared discoveries between each method and the proposed procedure across varying FDR thresholds in the BV case study. The denominator is the number of discoveries identified by each method. Similar to what we observed in simulations, not all discoveries identified by existing methods were declared significant by the proposed procedure. By accounting for the global structure of the graph, the new procedure interprets some extreme test statistics as noise rather than true signals, and consequently finds no evidence of a difference between BV and healthy individuals for those associations.

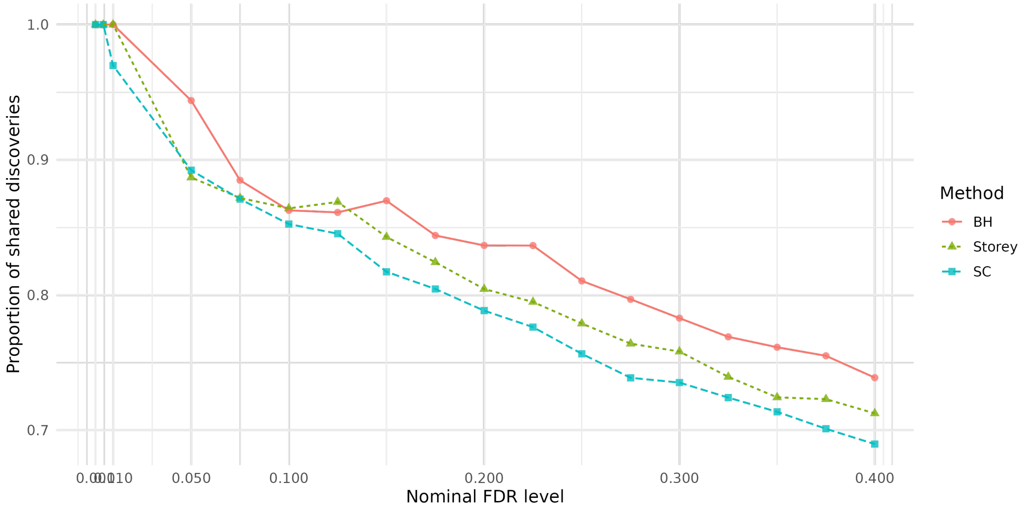

Web Figure 11: Proportion of shared discoveries between each method and the proposed procedure across varying FDR thresholds in the BV case study. The denominator is the number of discoveries identified by each method. Larger values indicate that more discoveries identified by existing methods were also declared significant by the new procedure.

Web Appendix C.3 Dataset-specific diagnostics

To assess whether the biSBM-based procedure is suitable for a given dataset, we recommend performing the following dataset-specific diagnostics

(i) evidence for bipartite block structure

(ii) stability and identifiability of the inferred partitions

(iii) posterior-predictive checks of the fitted marginal distribution compared to the observed test statistics

For (i), one can perform singular value decomposition of the test statistics matrix to check evidence for a bipartite block structure—a low-rank elbow in the singular values indicates plausible block structures. In Web Figure 12, we see that there is an elbow in the singular

values of test statistics in the BV data set, although it is not very sharp. For (ii), one can evaluate subsample clustering stability by running the same procedure on multiple row/column subsamples (Lee et al., 2011) (Table 3; Web Appendix C.4). This is similar in spirit to stability selection employed in high-dimensional regression or graphical modeling (Meinshausen and Bühlmann, 2010; Liu et al., 2010). For (iii), one can visualize the fitted marginal distribution against the empirical distribution of observed test statistics to assess goodness of fit (Figure 4A of the main text).

When these diagnostics indicate weak or unstable block structure—or strong deviations such as hub-dominated patterns or heavy degree heterogeneity—simpler mixture methods (e.g., SC) or alternative network priors (e.g., degree-corrected/hub models) are preferable.

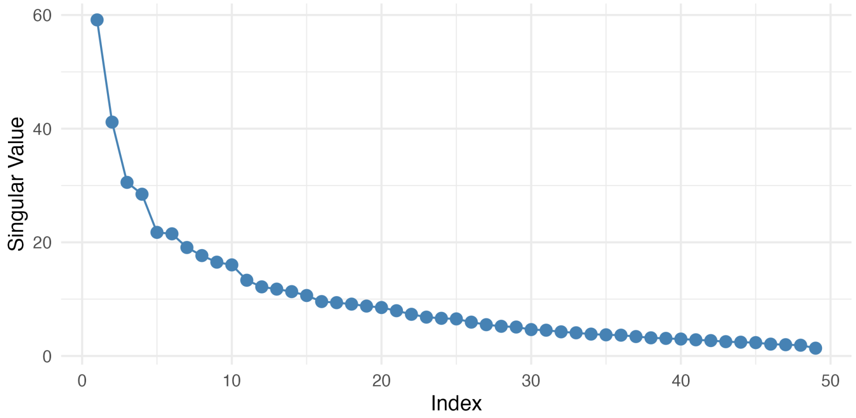

Web Figure 12: Singular values of test statistics in the BV case study.

Web Appendix C.4 Stability assessment of biclustering solutions

We evaluated the reproducibility of each biclustering solution using a subsampling-based stability procedure adapted from Lee et al. (2011). Rather than relying solely on internal fit criteria, stability analysis quantifies how consistently individual observations are assigned to the same bicluster across multiple perturbed datasets. High stability indicates that the inferred biclusters capture reproducible structure in the data rather than spurious patterns or noise.

Specifically, we repeatedly generated subsampled matrices by selecting a fraction f_r of rows and f_c of columns without replacement. For each subsample $b = 1, \dots, T$, we refit the biclustering algorithm to obtain a subsample-specific solution $M^{(b)}$. Across the T subsamples, we computed row and column co-membership probabilities:

$$C_{\text{row}}(i, i') = \frac{\#\{\, b : Z_1^{(b)}(i) = Z_1^{(b)}(i') \,\}}{\#\{\, b : i, i' \text{ both observed in subsample } b \,\}},$$

$$C_{\text{col}}(j, j') = \frac{\#\{\, b : Z_2^{(b)}(j) = Z_2^{(b)}(j') \,\}}{\#\{\, b : j, j' \text{ both observed in subsample } b \,\}},$$

where $Z_1^{(b)}$ and $Z_2^{(b)}$ denote the row and column cluster labels in the b-th subsample.

Let M denote the biclustering solution on the full dataset with K biclusters and M_k the k-th bicluster of M. Each bicluster in the full-data fit is a rectangular block $M_k = R_k \times C_k$, where R_k and C_k refer to the set of rows and columns belonging to M_k, respectively. Before computing bicluster agreement between the full-data and each subsample, the full-data biclusters were restricted to the rows and columns present in each subsample. We quantified stability of the row and column components via

$$c_{\text{row}}^{(k)} = \frac{1}{\binom{|R_k|}{2}} \sum_{\substack{i<i' \\ i,i' \in R_k}} C_{\text{row}}(i,i'), \qquad c_{\text{col}}^{(k)} = \frac{1}{\binom{|C_k|}{2}} \sum_{\substack{j<j' \\ j,j' \in C_k}} C_{\text{col}}(j,j').$$

Here $|R_k|$ denotes the size of the set R_k. A bicluster is stable only if both its row and column memberships are stable. Thus its overall stability is defined as the product

$$c_{\text{bic}}^{(k)} = c_{\text{row}}^{(k)} \cdot c_{\text{col}}^{(k)}.$$

Larger biclusters tend to exhibit inflated consensus due to chance alignment. To correct for this size-related bias, we computed a random baseline stability. For each bicluster M_k, we generated random biclusters of the same dimensions by permuting row and column indices and extracting blocks of sizes $|R_k|$ and $|C_k|$. Let $c_{\text{rand}}^{(k)}$ denote the average stability of such random blocks. We then defined a normalized bicluster stability measure following Lee et al. (2011):

$$\tilde{c}^{(k)} = \frac{c_{\text{bic}}^{(k)} - c_{\text{rand}}^{(k)}}{1 - c_{\text{rand}}^{(k)}}.$$

Values near 1 indicate high stability beyond chance, values near 0 indicate stability comparable to random partitions, and negative values indicate instability.

Finally, a model M with K biclusters $M_1, \ldots, M_K$ was summarized using a size-weighted normalized stability:

$$\tilde{S}_{\text{model}} = \frac{\sum_{k=1}^{K} (|R_k|\,|C_k|)\,\tilde{c}^{(k)}}{\sum_{k=1}^{K} (|R_k|\,|C_k|)}.$$

This index aggregates stability over all biclusters, weighting by the number of cells represented by each block. Models with higher $\tilde{S}_{\text{model}}$ were considered more reproducible under subsampling.

In the presence of strong and well-separated block structures, stability and ICL-based model selection criteria tend to yield consistent results (Web Table 4). In practice, data are often noisier and the underlying block structures are less well-separated. To avoid overfitting, we recommend picking the model that yields high stability and an ICL within 10-15% of the maximum. Web Table 3 summarizes the stability estimation results for a subset of models in the BV case study. The most reproducible bicluster structure was obtained with $Q_1 = 4$ and $Q_2 = 1$, despite its corresponding ICL value being lower than the maximum value of -22539.14.

Web Table 3: Stability estimation results for the ten models with the highest stability scores in the BV case study. A total of $T = 50$ subsamples were used, with subsampling fractions $f_r = f_c = 0.8$. *ICL* denotes the integrated classification likelihood criterion.

Q_1	Q_2	stability score	ICL
4	1	0.76	-25882.72
2	2	0.73	-26638.08
2	3	0.71	-26512.33
5	1	0.69	-26587.75
1	2	0.65	-27158.73
3	2	0.62	-26602.67
6	1	0.62	-27156.66
4	3	0.61	-25130.91
4	2	0.60	-25127.99
3	3	0.59	-25993.93

Web Appendix D Data and Code

The data set from McMillan et al. (2015) is publicly available at `https://doi.org/10.1038/srep14174`. An R package implementing the proposed method is available at `https://github.com/drjingma/metaMint`. Data and code used to reproduce results in this paper can be found at `https://github.com/drjingma/metaMint-paper-materials`. For the Sun & Cai procedure, we used the implementation publicly available at `http://www-stat.wharton.upenn.edu/~tcai/paper/html/FDR.html`.

References

Aitchison, J. and Bacon-Shone, J. (1984). Log contrast models for experiments with mixtures. *Biometrika* **71,** 323–330.

Cole, J. R., Wang, Q., Fish, J. A., Chai, B., McGarrell, D. M., Sun, Y., Brown, C. T., Porras-Alfaro, A., Kuske, C. R., and Tiedje, J. M. (2014). Ribosomal database project: data and tools for high throughput rrna analysis. *Nucleic Acids Research* **42,** D633–D642.

DeSantis, T. Z., Hugenholtz, P., Larsen, N., Rojas, M., Brodie, E. L., Keller, K., Huber, T., Dalevi, D., Hu, P., and Andersen, G. L. (2006). Greengenes, a chimera-checked 16s rrna gene database and workbench compatible with arb. *Applied and Environmental Microbiology* **72,** 5069–5072.

Larremore, D. B., Clauset, A., and Jacobs, A. Z. (2014). Efficiently inferring community structure in bipartite networks. *Physical Review E* **90,** 012805.

Lee, Y., Lee, J., and Jun, C.-H. (2011). Stability-based validation of bicluster solutions. *Pattern Recognition* **44,** 252–264.

Web Table 4: Stability estimation results for the ten models with the highest stability scores in one data set under Scenario (a) (Section 4). A total of $T = 50$ subsamples were used, with subsampling fractions $f_r = f_c = 0.8$. *ICL* denotes the integrated classification likelihood criterion. The model maximizing ICL coincides with the model maximizing stability, at $Q_1 = Q_2 = 3$.

Q_1	Q_2	stability score	ICL
3	3	0.99	-109460.31
3	4	0.92	-109493.29
4	3	0.92	-109671.35
5	3	0.83	-109755.22
3	5	0.82	-109975.83
4	4	0.81	-109621.10
4	5	0.73	-118662.96
5	4	0.73	-109871.43
2	2	0.70	-118231.76
4	2	0.67	-118687.71

Liu, H., Roeder, K., and Wasserman, L. (2010). Stability approach to regularization selection (stars) for high dimensional graphical models. *Advances in Neural Information Processing Systems* **23,**.

Ma, J. (2021). Joint microbial and metabolomic network estimation with the censored gaussian graphical model. *Statistics in Biosciences* **13,** 351–372.

McMillan, A., Rulisa, S., Sumarah, M., Macklaim, J. M., Renaud, J., Bisanz, J. E., et al. (2015). A multi-platform metabolomics approach identifies highly specific biomarkers of bacterial diversity in the vagina of pregnant and non-pregnant women. *Scientific Reports* **5,** 14174.

Meinshausen, N. and Bühlmann, P. (2010). Stability selection. *Journal of the Royal Statistical Society: Series B (Statistical Methodology)* **72,** 417–473.

Rebafka, T., Roquain, É., and Villers, F. (2022). Powerful multiple testing of paired null hypotheses using a latent graph model. *Electronic Journal of Statistics* **16,** 2796–2858.

Stein, S. E. (1999). An integrated method for spectrum extraction and compound identification from gas chromatography/mass spectrometry data. *Journal of the American Society for Mass Spectrometry* **10,** 770–781.

Styczynski, M. P., Moxley, J. F., Tong, L. V., Walther, J. L., Jensen, K. L., and Stephanopoulos, G. N. (2007). Systematic identification of conserved metabolites in gc/ms data for metabolomics and biomarker discovery. *Analytical Chemistry* **79,** 966–973.

Sun, W. and Cai, T. T. (2007). Oracle and adaptive compound decision rules for false discovery rate control. *Journal of the American Statistical Association* **102,** 901–912.

Yoon, G., Gaynanova, I., and Müller, C. L. (2019). Microbial networks in spring-semi-parametric rank-based correlation and partial correlation estimation for quantitative microbiome data. *Frontiers in Genetics* **10,** 516.
