# Supplementary material for: Inference for microbe–metabolite association networks using a latent graph model
Source: Biometrics. 2026 Mar 10;82(1):ujag042. doi: 10.1093/biomtc/ujag042 (PMC13017156; doi:10.1093/biomtc/ujag042)
Supplement: ujag042_Supplemental_Files — Web Appendices, Tables, Figures from additional simulation and case studies, and data and code for reproducing the results referenced in Sections 3–6 are available with this article at the Biometrics website on Oxford Academic. [file ujag042_supplemental_files.zip › code.zip!/metaMint-paper-materials/README.html]

README


# About

This repository provides code for reproducing analysis in the
metaMint paper.

## biSBM

This folder contains the R functions used in simulations and data
analysis. The code in the subfolder **FDR-R-Code/**
implements the Sun and Cai (2007) procedure.

## Simulations

This folder contains the R code used in simulations.
Specifically,

- `sim_Gaussian.R`: file used for simulating Gaussian
  distributed test statistics with a bipartite network generated under the
  noisy bipartite stochastic block model. This is the main file used for
  most of the simulations.
- `sim_Gamma.R`: file used for simulating Gamma
  distributed test statistics.
- `sim_Gaussian_pa.R`: file used for simulating Gaussian
  distributed test statistics with a bipartite network obtained under the
  preferential attachment model.
- `sim_Gaussian_nested.R`: file used for simulating
  Gaussian distributed test statistics with a nested bipartite
  network.
- `generate_correlation.R`: file to generate correlation
  matrix used to simulate multivariate data as done in
  `sim_mvdata.R`.
- `sim_mvdata.R`: file used for simulating data directly
  from multivariate distributions and computing test statistics
  afterwards
- `sim_stab.R`: file to perform cluster stability
  selection in simulated data.
- `sim_biSBM_SBM.R`: file to compare biSBM with the
  original SBM-based model
- `metrics_Gaussian.R`: file to summarize the simulation
  results when data are Gaussian distributed
- `metrics_Gamma.R`: file to summarize the simulation
  results when data are Gamma distributed
- `metrics_mvdata.R`: file to summarize the simulation
  results when data are multivariate
- `sim_Gaussian.sh`, `sim_Gamma.sh`,
  `sim_mvdata.sh` are script files used to submit jobs on the
  cluster. Arguments inside each file can be modified to change between
  settings. Use the command:

  ```
  sbatch sim_Gaussian.sh
  ```

The subfolder **data/** contains data needed in the
simulations.

- `20250507_n1_150_n2_200_Q1_2_Q2_2_Gauss01_setting5_network.rds`:
  bipartite network obtained with the preferential attachment model.
- `correlation_n1_49_n2_50_Q1_3_Q2_3.rds`: correlation
  matrices used in simulating multivariate data.

## Data

This folder contains data and code used in the analysis of the BV
data set.

- `BV.rda`: file containing the processed taxa relative
  abundances and metabolite concentrations.
- `BV_processing.R`: file used to process the data and
  obtain the file `BV.rda`
- `BV.R`: file used to run the new procedure on the BV
  data set with stability selection. The user can choose to run the script
  with model selection or on a specific model by toggling between lines 12
  and 13.
- `BV_visualization.R`: file used to summarize results
  and visualize the bipartite network.
- `get_BV_teststat.R`: file to obtain test
  statistics.
- `BV_teststatistics_correlation.rds`: file containing
  the test statistics used. In the list object, both `cl` and
  `pearson` refer to test statistics obtained using the Cai and
  Liu approach. The only difference lies in the constant used when
  transforming taxa relative abundances. A constant of 0.01 leads to the
  test statistics `cl` while a constant of 0.1 leads to the
  test statistics `pearson`. They differ slightly, but they
  lead to essentially the same inferred microbe–metabolite network.

  ```
  X <- malabutils::clr.epsilon(ASV,const = 0.1)
  ```
